# Combined use of tri-axial accelerometers and GPS reveals the flexible foraging strategy of a bird in relation to weather conditions

**DOI:** 10.1371/journal.pone.0177892

**Published:** 2017-06-07

**Authors:** Jesús Hernández-Pliego, Carlos Rodríguez, Giacomo Dell’Omo, Javier Bustamante

**Affiliations:** 1 Department of Wetland Ecology, Estación Biológica de Doñana (CSIC), Seville, Spain; 2 Department of Conservation Biology, Estación Biológica de Doñana (CSIC), Seville, Spain; 3 Ornis italica, Rome, Italy; Brown University, UNITED STATES

## Abstract

Tri-axial accelerometry has proved to be a useful technique to study animal behavior with little direct observation, and also an effective way to measure energy expenditure, allowing a refreshing revisit to optimal foraging theory. This theory predicts that individuals should gain the most energy for the lowest cost in terms of time and energy when foraging, in order to maximize their fitness. However, during a foraging trip, central-place foragers could face different trade-offs during the commuting and searching parts of the trip, influencing behavioral decisions. Using the lesser kestrel (*Falco naumanni*) as an example we study the time and energy costs of different behaviors during the commuting and searching parts of a foraging trip. Lesser kestrels are small insectivorous falcons that behave as central-place foragers during the breeding season. They can commute by adopting either time-saving flapping flights or energy-saving soaring-gliding flights, and capture prey by using either time-saving active hovering flights or energy-saving perch-hunting. We tracked 6 lesser kestrels using GPS and tri-axial accelerometers during the breeding season. Our results indicate that males devoted more time and energy to flight behaviors than females in agreement with being the sex responsible for food provisioning to the nest. During the commuting flights, kestrels replaced flapping with soaring-gliding flights as solar radiation increased and thermal updrafts got stronger. In the searching part, they replaced perch-hunting with hovering as wind speed increased and they experienced a stronger lift. But also, they increased the use of hovering as air temperature increased, which has a positive influence on the activity level of the preferred prey (large grasshoppers). Kestrels maintained a constant energy expenditure per foraging trip, although flight and hunting strategies changed dramatically with weather conditions, suggesting a fixed energy budget per trip to which they adjusted their commuting and searching strategies in response to weather conditions.

## Introduction

The application of technological advances is expanding the frontiers of knowledge and opening new perspectives in ecological studies on free-ranging animals. The ongoing miniaturization and sophistication of tracking devices has broadened the range of species that can be studied with unprecedented spatial and temporal resolution [1,2]. In the light of this recent technological revolution, the number of studies on animal movement has increased and, with it, the need to create a framework to encompass them all. This has been the breeding ground for the Movement Ecology paradigm [3]. This paradigm advocates that individual movement results from the interaction between individual internal state, motion and navigation capacities, and external factors. Apart from tracking devices, a series of animal-borne sensors or biologgers are being developed that are helping us to understand the factors that determine the movement path, including accelerometers that are starting to be one of the devices most widely used nowadays.

Accelerometers measure body acceleration across one, two or three spatial axes at high temporal resolutions (typically 10 Hz or more). These devices inform about animal body position via the static component of acceleration that indicates device, and hence body orientation, with respect to the Earth’s gravitational field [4,5]. Accelerometers also allow researchers to deduce animal behavior through the dynamic component of acceleration that results from the inertia created when animal body moves [6,7]. Therefore, accelerometers help disentangling how free-ranging animals adjust behaviors in time (and also in space when coupled with tracking devices) with no need or reduced direct observation in the field. Consequently, they reduce observer bias and save working time and effort [8,9]. In addition, accelerometry has been proved to be a useful technique to measure animal energy expenditure related to locomotion [10,11]. Animals require energy to perform their behaviors, each characterized by specific three-dimensional body movement. So it has been hypothesized that animal body movement would be proportional to the energy invested in producing it. Wilson et al. [12] demonstrated that body acceleration correlates well with oxygen consumption in great cormorants *Phalacrocorax carbo* when walking at different speeds on a treadmill. Since then, several studies have come to the same conclusion using a variety of study species moving freely across land, air or water [13–15]. Tri-axial accelerometry has been used with different purposes, such as identifying hidden or anomalous behaviors and analyzing daily activity budget, but perhaps it has found its main application in the study on foraging behavior [16–22]. The reason might be related to the fact that accelerometers provide simultaneous information about the energy and time budget of wild animals, which is of paramount importance under the framework of the optimal foraging theory [23].

The optimal foraging theory predicts that individuals should modulate their foraging strategies to maximize their fitness by gaining the most energy at the lowest cost [23]. However, individual requirements may vary with dynamic endogenous (e.g. age, body condition, breeding status) and exogenous factors (e.g. prey availability, intraspecific competition, wind conditions) that shape their foraging strategies in space and time [24–29]. Central-place foragers are considered good models to test predictions derived from the optimal foraging theory since it is possible to separate the cost of travel between the central-place and the foraging patch from that of resource acquisition at the foraging patch during the foraging trip [30]. Central-place foragers usually experience different conditions when commuting versus searching for food that leads them to behave differently in order to deal with those challenges along the foraging trip. For example, northern gannets *Morus bassanus* leave the breeding colony flying with the wind in order to reduce flight cost when commuting to foraging patches, whereas at the foraging patch they fly against the wind presumably to increase prey detection by reducing flight ground speed [31]. Therefore, central-place foragers may face different trade-offs when commuting than when searching for food that could influence their behavioral decisions along the path, and ultimately determine the overall cost of foraging trips.

In this paper, we studied the foraging behavior of the lesser kestrel (*Falco naumanni*) during the breeding season. Lesser kestrels behave as central-place foragers because breeding individuals fly from the colony to the foraging patch where they capture prey and return to the colony carrying a single prey item in their beak or talons. They can fly between the colony and the foraging patches by using either flapping or soaring-gliding flights [32]. Birds flying with flapping flights transform chemical energy at their muscles to beat their wings gaining mechanical energy. Meanwhile, birds using soaring-gliding flights harvest kinetic energy from the atmosphere, mainly from uprising thermal air currents. Thus, flapping flights are more energy-consuming than soaring-gliding flights, but birds fly at higher cross-country speeds with flapping flights compared to those attained with soaring-gliding flights [33]. At the foraging patch, kestrels can capture prey either by hovering flights (an active hunting strategy in which kestrels remain suspended in the air flapping their wings) or from a perch (a passive sit-and-wait hunting strategy from an elevated position) [34]. The active hunting strategy involves that the kestrel flies continuously with eventual hovering bouts while searching for prey with an associated high energy expenditure. By contrast, in the sit-and-wait hunting strategy the kestrel waits from a perch until a prey enters its field of vision and then flies and attempts a capture. Therefore, the active hunting strategy requires more energy per time unit than the sit-and-wait hunting strategy, but the former is more time-efficient for finding prey [35–37]. In this study, we investigate the influence of internal (phenological period, sexual role specialization) and external factors (weather conditions) on lesser kestrel behavioral decisions. We tracked lesser kestrel individuals from two colonies using combined GPS and tri-axial accelerometers during the breeding season. We identified and classified lesser kestrel behaviors in order to determine individual energy and time budget at three hierarchical levels of analyses: the day, the foraging trip, and the foraging trip segment (distinguishing between commuting flights and foraging event). First, we analyzed the effect of sex and phenological period on the lesser kestrel’s daily energy and time budget, because it is known that these variables influence strongly lesser kestrel movements [38–40]. Second, we analyzed the effect of time of day on foraging trip energy and time budget to unravel how lesser kestrels distribute their energy and time available in foraging effort along the day. Finally, we analyzed how lesser kestrels adapt their flight (flapping versus soaring-gliding) and hunting strategies (hovering versus perching) during the commuting flights and the foraging event segments of the foraging trip, respectively, to weather conditions (wind speed, air temperature, solar radiation, and rainfall).

## Material and methods

### Ethics statements

The environmental authority (Dirección General de Gestión del Medio Natural y Espacios Protegidos, Junta de Andalucía) provided permits to access the study colonies and to attach dataloggers to this endangered species. The Estación Biológica de Doñana Ethics Committee on Animal Experimentation (CEEA-EBD), the Bioethics Subcommittee of the Consejo Superior de Investigaciones Científicas (CSIC) and the Consejería de Agricultura, Pesca y Desarrollo Rural (Junta de Andalucía) all reviewed the marking protocol and approved the research plan of the HORUS project.

### Study species and area

The lesser kestrel is one of the smallest raptors in the Palearctic (wingspan 58–72 cm, body mass 120–140 g) and its diet is mostly based on large insects. This hole-nesting species breeds colonially in buildings and cliffs associated with steppe-like habitats, pastures, and non-irrigated crops across the Mediterranean basin and Central Asia, and it has its wintering grounds in Africa [41,42]. However, the world population has apparently stabilized in the last decades [43].

The study was carried out at two breeding colonies of lesser kestrels (“Silo” and “EBD” colonies) located in the Guadalquivir river basin (southwestern Spain), which is predominantly flat (20–240 m above sea level) and dominated by arable crops [44]. Primary crops are wheat and sunflowers, although cotton, legume crops, olive groves and vineyards are also present in the area. The Silo colony is situated at a building with a grain elevator located in agricultural land, while the EBD colony is situated 50 km away on the roof of our research institute within the urban landscape of the city of Seville. Kestrel pairs breed inside nest-boxes installed at both buildings.

### Device deployment

We deployed a GPS-datalogger (GiPSy-5 model, Technosmart, Italy) and a tri-axial accelerometer-datalogger (Axy-3 model, Technosmart, Italy) on lesser kestrel breeding adults monitored during the 2014 breeding season. The two tags were attached together on a carbon fiber plate and fixed to the birds’ backs using a 4 mm wide Teflon ribbon (Bally Ribbon Mills, Pennsylvania, U.S.A.) (S1 Fig). Before deploying, the devices were protected with a heat-shrinking tube. The total mass of the equipment (harness + tags) was about 6 g and never exceeded the 5% of the lesser kestrel’s mean body mass, which is within the generally recommended limits for flying animals [45]. We attached a plasticine dummy similar in size and weight to the devices to habituate birds to wear the harness and the devices at least a week prior to the deployment of the instruments (see details of the procedure in Hernández-Pliego *et al*. [39]).

We deployed the devices on six lesser kestrel breeders (four males and two females) (Table 1). We configured the GPS devices at two different sampling frequencies: 1 fix/sec (that gives a very detailed track) and 1 fix/3 min (that maximizes battery duration). GPS provided spatial location, altitude and instantaneous speed. We configured the accelerometer devices to record acceleration at 10 Hz on three axes: the kestrel’s antero-posterior axis (surge, X), the lateral axis (sway, Y) and the dorso-ventral axis (heave, Z). The activation of the accelerometer was done at a precise GPS time so that the two instruments were synchronized. Since the GPS and the tri-axial accelerometers stored the data in loggers, we had to recapture the individuals to recover the data. A new full-powered set of devices was then deployed before releasing the individual to resume tracking. Kestrels were captured when they entered the nest-boxes using remote-controlled sliding doors. Individuals were recaptured a mean of 2 times during the study period (range 1–3, n = 6). GPS operated during daylight to save battery (5 to 20 UTC), while accelerometers recorded data continuously during the entire day. Data collection ranged from 3^rd^ June to 24^th^ June 2014. At some nests kestrels where incubating while at others eggs had started to hatch, so our data were collected during the incubation and the nestling periods. We removed the harnesses from the kestrels at the end of the breeding season. GPS data from the study were stored in Movebank (http://www.movebank.org) [46].

**Table 1:** Details of individual lesser kestrels tracked during the study period.

Individual ID	Sex	Phenological Period	Breeding Colony	# Complete Days (3-min)	# Foraging Trips (1-sec)	# Foraging Trips (3-min)	Date of First Deployment	Hatching Date	Clutch Size
B[6.U]	Male	Incubation	Silo	3	0	6	03/06/2014	11/06/2014	1
B[6.U]	Male	Nestling	Silo	2	0	25	03/06/2014	11/06/2014	1
B[D.A]	Female	Nestling	Silo	6	4	14	09/06/2014	04/06/2014	4
B[H.J]	Male	Incubation	Silo	0	0	5	03/06/2014	04/06/2014	4
B[H.J]	Male	Nestling	Silo	10	18	202	03/06/2014	04/06/2014	4
B[H.Y]	Male	Incubation	Silo	6	0	9	03/06/2014	10/06/2014	3
B[H.Y]	Male	Nestling	Silo	0	6	0	03/06/2014	10/06/2014	3
V[0A7]	Male	Nestling	EBD	5	0	118	11/06/2014	04/06/2014	3
V[0AF]	Female	Nestling	EBD	3	0	37	17/06/2014	04/06/2014	3

### Weather data

We obtained wind speed, air temperature, solar radiation and rainfall data from the agroclimatic weather station network of the Andalusian Agricultural Department (http://www.juntadeandalucia.es/medioambiente/servtc5/WebClima/), collected at the meteorological station of La Palma del Condado by a RM Young 05103 windmill anemometer, a Vaisala HMP45C temperature sensor, a Skye SP1110 pyranometer and a Campbell ARG100 pluviometer, respectively. All weather variables were sampled every 30 minutes. The station is situated 192 m above sea level, 3 km from the Silo colony and 48 km from the EBD colony.

### Analytical procedures

**Fig 1:**
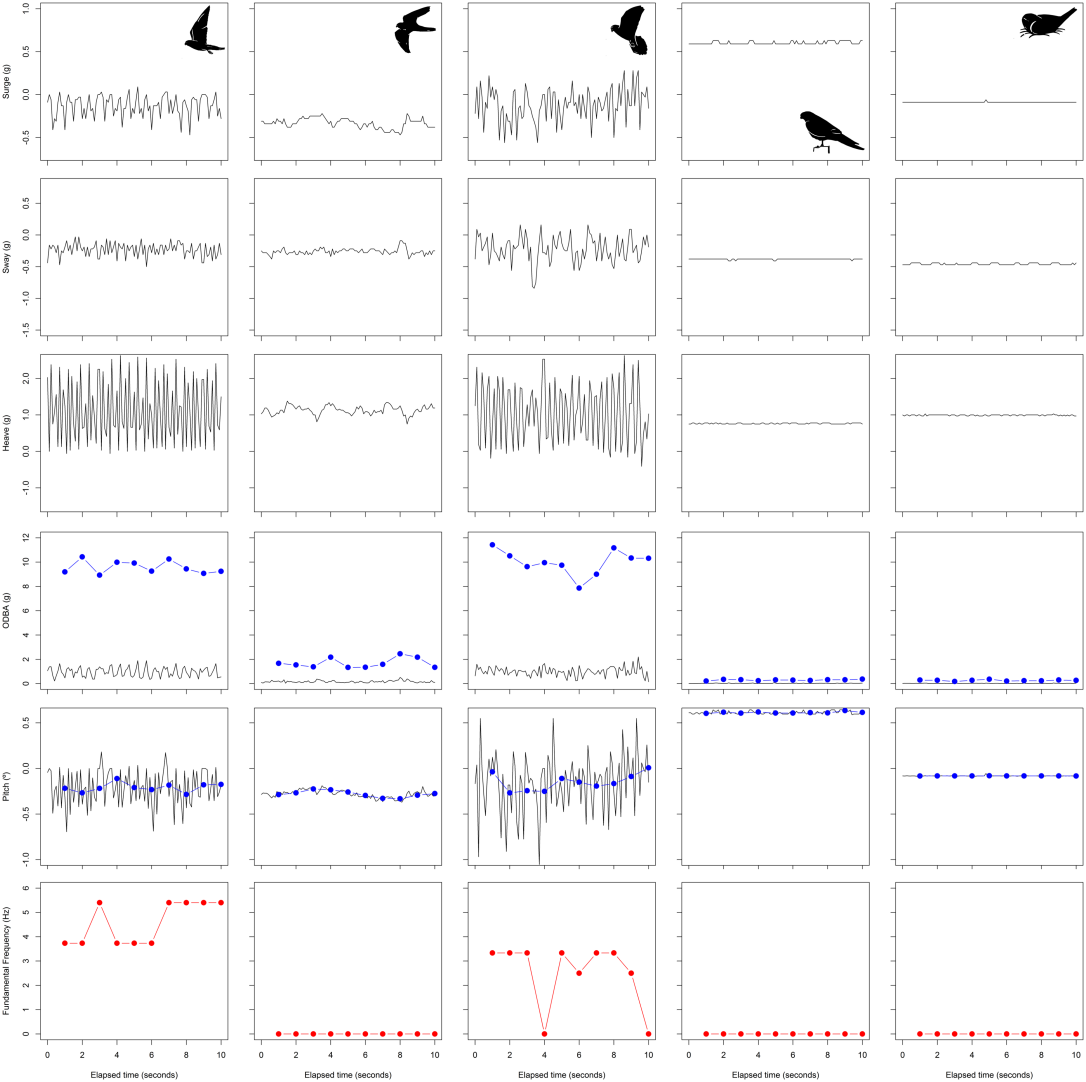
Acceleration signature of different lesser kestrel behaviors. Raw acceleration measured in the surge (X), sway (Y) and heave (Z) axes, ODBA, pitch, and fundamental frequency of the heave axis (rows) in flapping flight, soaring-gliding flight, hovering flight, perching, and incubating/brooding behaviors (columns). Blue dots indicate ODBA and mean pitch at 1-second interval in their respective panels. Red dots indicate fundamental frequency of the heave axis at 1-second interval.

High-frequency GPS fixes (1 fix/sec) allowed us to distinguish unequivocally if a kestrel was flying or stationary by using instantaneous speed and relative spatial position of fixes. Additionally, accelerometer signature allowed us to know which type of flight was adopted by the kestrel when flying, and whether kestrels were perching when stationary. We identified three flight behaviors (flapping, soaring-gliding, and hovering) and two stationary behaviors (perching and incubating/brooding) based on data from three individuals (two males and one female) tracked with the GPS at 1 fix/sec (Fig 1). Individuals mostly used flapping and soaring-gliding flights along commuting segments of the foraging trip when moving between the colony and the foraging area. Hovering flights are the main hunting strategy for kestrels (active hunting), therefore this flight behavior appeared exclusively during the foraging event. Kestrels also hunt from a perch (sit-and-wait strategy). Thus, perching behavior recorded during the foraging events was considered to be perch-hunting, but when associated with the colony or roosts was considered resting behavior. Incubating/brooding behavior was only adopted at the colony while incubating eggs or brooding chicks. We used 1-s intervals of acceleration data, i.e. 10 consecutive acceleration measures, as the minimum sample unit to label behaviors. Flapping and hovering flights were characterized by regular oscillations in the surge (X) and heave (Z) axes due to wing beats, but the former was associated with GPS instantaneous speed higher than 0.5 m/s whereas the latter was associated with speeds below 0.5 m/s (as kestrels remain suspended in the air while hovering). Soaring-gliding flight was differentiated from flapping flight because of the absence of a regular oscillation in any axis. Similarly, GPS instantaneous speed allowed us to distinguish between soaring-gliding flight (with speeds higher than 0.5 m/s) and stationary behaviors (with speeds below 0.5 m/s). Within stationary behaviors, perching showed positive values in the surge (X) axis, whereas incubating/brooding showed values around zero in this axis because of the different angle of the body between these two behaviors (Fig 1). A similar algorithm for assigning bird behavior from accelerometer signature has been proposed in previous studies [47,48]. We manually labeled behaviors by analyzing 184 min of acceleration data gathered from the foraging trips of one female, and 83 min and 79 min of two males. Then, we trained a classification model in order to automatically classify behaviors from accelerometer data following the procedure described in Shamoun-Baranes *et al*. [49]. We used decision trees as the learning method of the model. We selected at random 70% of the labeled acceleration data to train the model leaving the remaining 30% to test it. We tested as predictors 18 variables derived from acceleration data: mean value and standard deviation of acceleration in each of the three axes, plus pitch and roll, pairwise correlation between the axes, fundamental frequency of acceleration cycles in the three axes, overall dynamic body acceleration (ODBA) and vectorial dynamic body acceleration (VeDBA) (see Shamoun-Baranes *et al*. [49]). Each variable was calculated for every 1-s interval of acceleration data. Despite GPS instantaneous speed is widely used as a predictor of behavior in classification models [31,49], we did not include it in our model because we did not have instantaneous speed measures associated with every 1-second interval of acceleration data, because most GPS were programmed with a fix every 3 min to save battery. Including instantaneous speed as a model predictor would have prevented us from applying that model to automatically classify behaviors using only the acceleration data. As a consequence, soaring-gliding flight and incubating/brooding behavior were initially misclassified since both showed similar acceleration signatures on the three axes. In order to solve this problem, we carried out *a posteriori* classification to tag those 1-s intervals labeled initially as soaring-gliding as incubating/brooding: if the GPS location closest in time situated the individual kestrel at the breeding colony (300-m radius), if the instantaneous speed was below 0.5 m/s, and if the acceleration time series revealed that the individual was still stationary. We used this final model to classify behaviors using only the accelerometer data from all six individual lesser kestrels tracked, regardless of the GPS sampling frequency.

We evaluated the classification efficiency of the final model using a jack-knife procedure, building a model with data of two kestrels and classifying the behaviors of the third, and repeating this procedure until all three kestrels had been used as test individuals. Furthermore, we carried out an extra validation of the final classification model using nighttime data between 21 p.m. to 4 a.m., when individuals are supposed to be resting, in order to test the percentage of correct classification of stationary behaviors. Nest-boxes from the Silo colony are equipped with video cameras (analog camera KPC-EX500B with an IR illuminator and a Vivotek 8102 video server) that record 10-second video sequences activated by movement detection [50]. In order to validate the decision rule to classify the incubating/brooding behavior using instantaneous speed and distance to the colony using the GPS position closest in time (1 fix/3 min), we randomly sampled 25 intervals classified as incubating/brooding per day from individuals breeding at the Silo colony, then we cross-classified this information with what could be observed on the video samples from the corresponding nest-box.

### Activity budget variables

GPS data were explored graphically using GIS (ArcGIS 10, ESRI, Redlands, California, U.S.A.) to identify the foraging trips. We use the term foraging trip to refer to a set of consecutive locations of a kestrel that, starting from the breeding colony, go farther than 300 m, and in which we are able to identify a foraging event (mostly clumped locations at low altitude above the ground with highly variable instantaneous speed). We segmented every foraging trip in three parts: (1) the outward flight, i.e. the movement from the colony or roost to the foraging area; (2) the foraging event, i.e. the movement within the foraging area; and (3) the inward flight, i.e. the return from the foraging area to the colony or roost. We considered as foraging event the segment of the foraging trip between the first and last hovering or perching bout identified along the trip. Therefore, the outward flight is the foraging trip segment before the first hovering or perching bout, whereas the inward flight is the foraging trip segment after the last hovering or perching bout. The outward and inward flights are the two types of commuting flights of the foraging trip. We considered as hovering or perching bout a sequence of at least five 1-s intervals of acceleration data labeled as hovering or perching, respectively. If two hovering bouts or two perching bouts were interrupted by less than 5 seconds of another behavior, we considered them as a single hovering or perching bout.

We estimated individual energy and time devoted to each behavior at three hierarchical levels: the day level, the foraging trip level, and the foraging trip segment level. As individual energy, we used ODBA at all levels of analyses since it can be taken as a proxy for energy expenditure [12]. ODBA per 1-s interval was calculated by summing the dynamic accelerations measured on the three orthogonal axes in each interval. Dynamic accelerations were obtained by subtracting the static component of the acceleration from the raw acceleration data, which was obtained by calculating the mean value of raw acceleration data in each 1-s interval. At the day level, we only included in the analyses complete days of tracking that are those in which we obtained 24 hours of continuous acceleration data. At the foraging trip level, we calculated the maximum distance from the colony and the duration of the foraging trip. Incomplete foraging trips, i.e. trips in which departure from or arrival at the colony or roost was not recorded by the GPS were removed from the analyses. At the foraging trip segment level, we calculated the duration of the three foraging trip segments (outward and inward commuting flights, and the foraging event). Moreover, we calculated the proportion of time and energy spent in each flight strategy (flapping versus soaring-gliding) during the commuting flights and the proportion of time and energy spent in each hunting strategy (hovering versus perch-hunting) during the foraging event. We calculated a flapping ratio as the time devoted to flapping flight divided by the total time spent in flight behaviors during commuting flights. Similarly, we calculated a hovering ratio as the time devoted to hovering flight divided by total time spent in hunting behaviors during the foraging event. In addition, since kestrels capture a single prey item per foraging trip when provisioning the nest, we estimated the foraging efficiency through calculating the number of hovering and perching bouts per foraging event. However, since kestrels may perch after capturing prey in order to eat it [51], we considered that kestrels were feeding themselves when hovering bouts were followed by a perching bout. We calculated the number of these “hovering-perching bouts” per foraging event.

### Statistical analysis

We evaluated the influence of sex and phenological period on the energy and time activity budget at the day level. We assessed the effect of the time of day on the kestrel energy and time activity budget at the foraging trip and foraging trip segment levels. We also analyzed the influence of weather (wind speed, air temperature and solar radiation) on the flight and hunting strategies of the lesser kestrel at the foraging trip segment level. The effect of rainfall could not be tested because there was no rainfall in 98.20% of the samples (n = 1056).

At the day level, we fitted Generalized Linear Mixed Models (GLMMs) to total ODBA (a proxy for energy expenditure) and to percentage of ODBA and percentage of time devoted to each behavior (energy and time allocation) per day. We included the individual identity and the breeding colony as random factors in the models. We included individual sex as a categorical predictor with two levels (male and female), because raptors are mostly a role-specialized group [52], and sex can have a strong influence on individual behavior. We also included phenological period as a categorical predictor in the models with two levels (incubation and nestling periods) because individuals have different energy demands and behave differently when incubating the eggs or raising the chicks.

At the foraging trip level, we fitted Generalized Additive Mixed Models (GAMMs) to total ODBA, percentage of ODBA and percentage of time devoted to each behavior, trip duration and trip maximum distance from the colony. In order to test the presence of a circadian pattern in lesser kestrel flight and hunting strategies we included hour-of-day (at the time foraging trip started rounded to the nearest half-hour) as a continuous predictor in these models. Kestrels have already shown a marked circadian pattern in soaring behavior [38]. We included individual identity and breeding colony as random factors in these GAMMs. Sex and phenological period were also included as categorical predictors in all models as they might have important influence on the variables analyzed at this level of analysis (see previous paragraph).

At the foraging trip segment level, we fitted GAMMs to ODBA and duration. In commuting flight segments, we also modeled the flapping ratio as a response variable. And in foraging event segments, we also modeled the hovering ratio, the number of hovering bouts, the number of perching bouts, and the number of hovering-perching bouts per foraging event as response variables. We included hour-of-day (at the time segment started rounded to the nearest half-hour) as a continuous predictor in the models. We also included commuting flight type as a categorical predictor with 2 levels (outward or inward flight) to assess potential differences in flight behavior of kestrels when leaving or returning to the colony or roost. In the models fitted to flapping and hovering ratio we also included wind speed and solar radiation or air temperature as continuous predictors, which were measured at the time (rounded to the nearest half-hour) when the foraging trip segment started. We did this in order to evaluate the influence of weather variables on kestrel flight and hunting behavioral decisions throughout the day (S2 Fig). Air temperature and solar radiation are moderately correlated (R^2^ = 0.50, n = 1056). To avoid collinearity, we did not include both variables as predictors in a single model. Consequently, we built two alternative models for both flapping and hovering ratio, each of which included either air temperature or solar radiation. They were subsequently compared to each other on their predictive ability. The weather predictor included in the best model of these two was also included in the final model for each response variable (AIC criteria, see model selection later in this section). We included individual identity and breeding colony as random factors in all models. Sex and phenological period were also included as categorical predictors in all GAMMs fitted at this level of analysis.

Percentage of ODBA and percentage of time devoted to each behavior were arcsine-square-root-transformed to meet the normality assumptions of generalized models, as is common for variables measured as percentages. Foraging trip ODBA and duration were logarithmically transformed to meet the generalized model assumption of residual homocedasticity. Flapping and hovering ratio were logit-transformed for the same reason. We used a Gaussian distribution of errors and the identity link function in most models. The only exceptions were foraging trip maximum distance from the colony and foraging trip segment ODBA and duration in which we used a gamma distribution of errors and a logarithmic link function (which were found to be more adequate after exploration of model residuals). To account for a potential nonlinear response to the predictor, we applied penalized smoothing splines to the hour-of-day, wind speed, air temperature and solar radiation in GAMMs. The degrees of freedom of the smoothing function were automatically selected using restricted maximum likelihood (REML) [53]. We followed the Akaike’s Information Criterion (AIC) for model selection (being the best model the one with the lowest AIC value). The best GAMMs for foraging trip ODBA, foraging event ODBA, and flapping ratio were those including the linear effect of the predictor, so we fitted a GLMM to those response variables using the same predictors and random factor. We fitted the GLMMs following a backward-stepwise procedure, by removing non-significant predictors until only significant ones remained. The significance of the predictors was tested using likelihood ratio tests comparing the model with and without the predictor.

Statistical analyses were performed using R-3.0.2 software [54]. We fitted GAMMs and GLMMs using “mgcv” [55] and “lme4” packages [56], respectively.

## Results

Models describing energy expenditure in behaviors by kestrels (ODBA) were analogous, in most cases, to models for time expenditure. Therefore, we usually show here the results of the models for time expenditure, and relegate the results of the models for energy expenditure in supplementary material.

### Behavioral identification from tri-axial accelerometers

**Fig 2:**
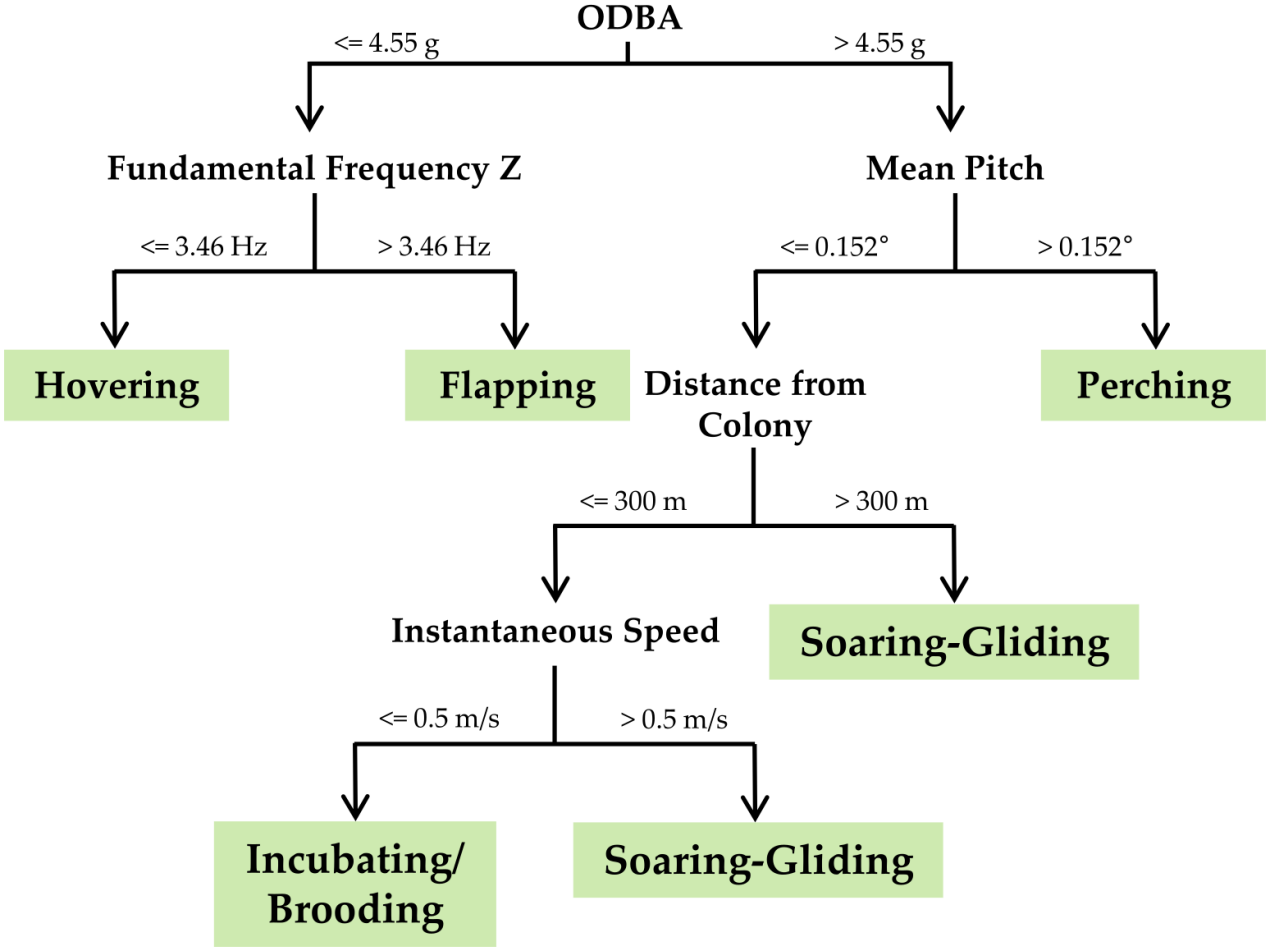
Decision tree for the final behavior classification model.

The final model to classify lesser kestrel behaviors (flapping, soaring-gliding, hovering, perching, and incubating/brooding) included as predictors the ODBA, the mean pitch, and the fundamental frequency of the heave axis per 1-s interval of acceleration data (Fig 1), as well as the instantaneous speed and the distance from the colony provided by the GPS (Fig 2, Table 2). The model showed 95% accuracy (Kappa = 0.93), indicating a reliable classification of behavior with low classification error (Table 3). The jack-knife testing (using two individuals to build the model and the third one to test it) also indicated a good model performance. This suggests that our model can be safely used to classify behaviors for individuals with no training data. The jack-knife validation is slightly worse than the validation with a random sample of all individuals, indicating that it is always better, when possible, to train the classification model with samples from the same individual (S1 Table). Validation of stationary behaviors (perching, and incubating/brooding) with nighttime video data resulted in, on average, 98.83 ± 2.76% of accuracy (n = 635,252 intervals). The validation using video sequences of the classification rule to distinguish between incubating/brooding and soaring-gliding (using distance to the colony and closest-in-time GPS speed) showed a mean accuracy of 77.33 ± 20.03% of incubating/brooding behavior (n = 675 intervals).

**Table 2:** Mean and standard deviation of raw acceleration measured in the surge (X), sway (Y) and heave (Z) axes, ODBA, pitch and fundamental frequency of heave axis per lesser kestrel behavior. Sample size = 3,024,000 intervals from six individual lesser kestrel (35 complete days of tracking).

Behavior	Surge (g)	Sway (g)	Heave (g)	ODBA (g)	Pitch (°)	Fundamental Frequency Heave (Hz)
Perching	0.54 ± 0.16	-0.02 ± 0.16	0.75 ± 0.12	0.4 ± 0.06	0.62 ± 0.21	0.004 ± 0.001
Incubating/brooding	-0.004 ± 0.17	-0.07 ± 0.16	0.90 ± 0.12	1.1 ± 0.14	-0.004 ± 0.19	0.003 ± 0.001
Soaring-gliding	-0.10 ± 0.12	-0.16 ± 0.14	0.98 ± 0.27	2.9 ± 0.25	-0.10 ± 0.14	0.003 ± 0.001
Flapping	-0.02 ± 0.21	-0.11 ± 0.22	1.00 ± 0.80	9.2 ± 0.47	-0.02 ± 0.27	4.01 ± 0.94
Hovering	0.04 ± 0.33	-0.08 ± 0.29	0.94 ± 0.79	9.5 ± 0.61	0.04 ± 0.40	3.15 ± 0.63

**Table 3:** Confusion matrix for the final classification model of behaviors. We built this matrix using the 30% of the tagged acceleration data selected at random to test the model after training it with the remaining 70%. Soaring-gliding and incubating/brooding are indicated as Gliding and Incubating, respectively. Observations correctly classified per behavior are shown in bold.

		Predicted Behaviors					
**Actual Behaviors**		Flapping	Gliding / Incubating	Hovering	Perching	Total	**Recall**
	Flapping	**934**	16	15	1	966	97%
	Gliding / Incubating	7	**1,257**	2	12	1,278	98%
	Hovering	46	12	**205**	1	264	78%
	Perching	3	36	3	**751**	793	95%
	Total	990	1,321	225	765	3,301	**Mean Recall** = 92%
	**Precision**	94%	95%	91%	98%	**Mean Precision** = 95%	**Accuracy** = 95% **Kappa** = 0.93

### Daily level

We recorded 35 days with 24-h accelerometer data, a mean of 5.83 ± 2.32 days per individual kestrel. Model for daily ODBA (energy expenditure) showed a statistically significant effect of phenological period and sex (Table 4). Kestrel males spent more energy than females, and they also showed higher daily energy expenditure during the nestling period versus the incubation period. We also found statistically significant effects of phenological period and sex on time and energy expenditure per behavior during daylight hours (Table 5, S2 Table). Kestrels dedicated on average 53.20 ± 22.55% of daylight hours to stationary behaviors (perching and incubating/brooding). Perching behavior was recorded equally frequent at the colony (50.69 ± 29.02%) than out of the colony (49.31 ± 29.02%) regardless the sex and the phenological period. Nevertheless, individuals allocated a higher proportion of daylight hours to incubate eggs during the incubation period than to brood chicks during the nestling period, and this behavior was less frequent in males than in females. Kestrels devoted on average 46.85 ± 22.55% of daylight hours to fly (flapping, soaring-gliding, and hovering flight behaviors). The time spent in flapping and in soaring-gliding flights during the daytime was shorter during the incubation period than during the nestling period and both time allocations were higher in males than in females. However, the time spent in hovering flights during the daylight hours was affected neither by phenological period nor by sex (Table 5). During the nighttime, kestrels allocated on average 98.12 ± 2.31% to stationary behaviors.

**Table 4:** Estimates (β), standard error (S.E.) and statistical significance of predictors included in the GLMM fitted to daily ODBA (energy expenditure per day) of the lesser kestrel. Statistically significant variables are shown in bold. Sample size = 35 complete days of tracking.

Predictors	β	S.E.	χ^2^	p-value
Intercept	204,961	15,959	-	-
**Sex** (Female)	- 64,552	25,654	3.96	**0.05**
**Phenological Period** (Incubation)	- 101,790	22,456	13.18	**< 0.001**

**Table 5:** Estimates (β), standard error (S.E.) and statistical significance of predictors included in the GLMM fitted to daily time expenditure in different behaviors by the lesser kestrel. Statistically significant variables are shown in bold: * p < 0.5, ** p <0.01, *** p<0.001. Sample size = 35 complete days of tracking.

Predictors	Intercept	Sex (Female)	Phenological Period (Incubation)
Behaviors	β ± S.E. (%)	β ± S.E. (%)	β ± S.E. (%)
Flapping	22.13 ± 0.08	- 14.68 ± 0.20 *	- 21.95 ± 0.15 ***
Soaring-Gliding	37.33 ± 0.38	- 16.22 ± 0.13 *	- 28.36 ± 0.14 ***
Hovering	5.08 ± 0.03	- 2.47 ± 0.09	- 2.51 ± 0.05
Perching	29.58 ± 0.06	13.75 ± 0.17	7.01 ± 0.16
Incubating/brooding	3.43 ± 0.23	10.21 ± 0.41 *	35.02 ± 0.35 ***

### Foraging trip level

**Fig 3:**
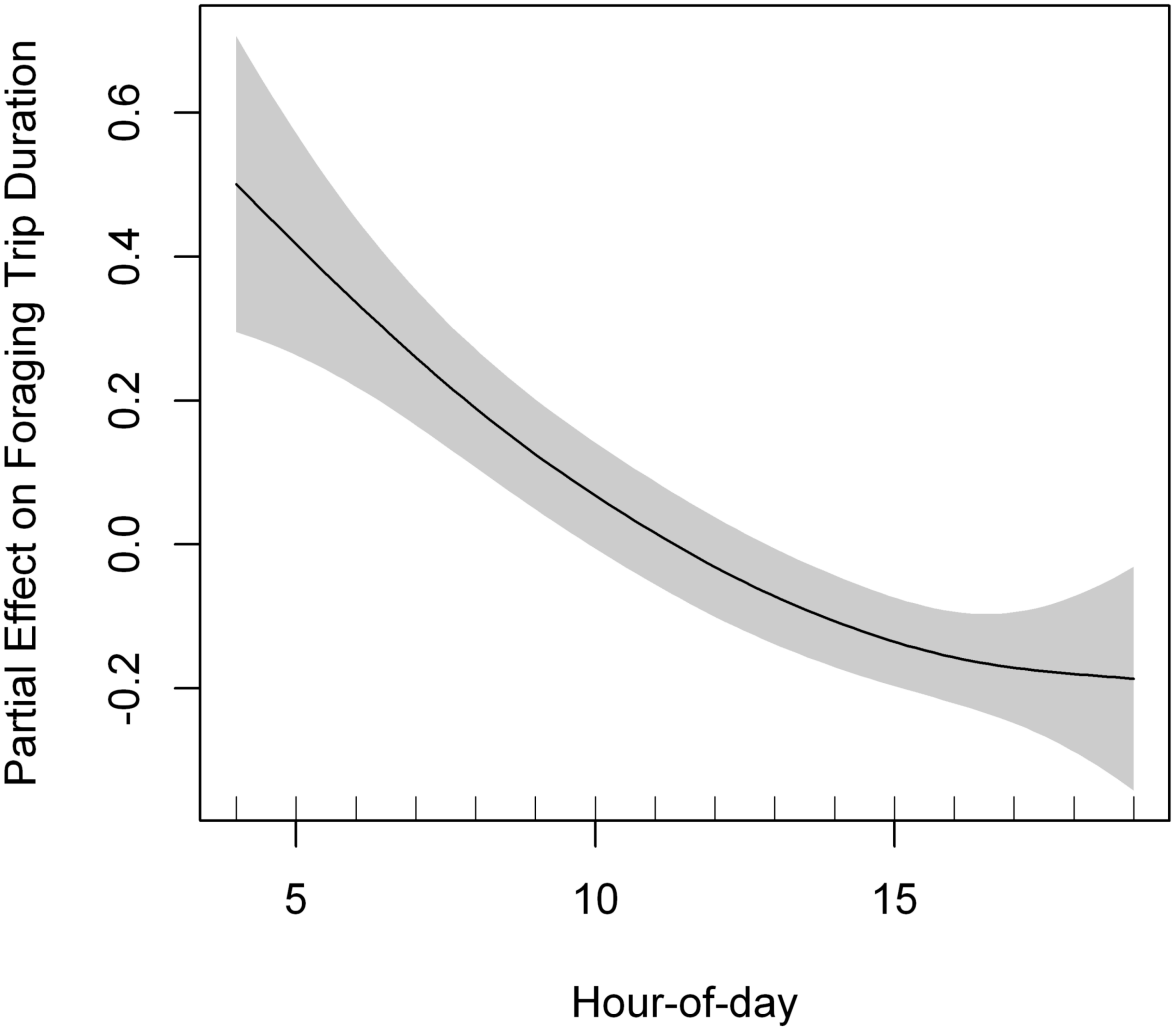
Partial effect of hour-of-day in the model fitted to lesser kestrel foraging trip duration. A penalized smoothing spline of 2.15 degrees of freedom was adjusted to hour-of-day. Grey shading represents the standard error of the mean effect. Sample size = 444 foraging trips.

**Fig 4:**
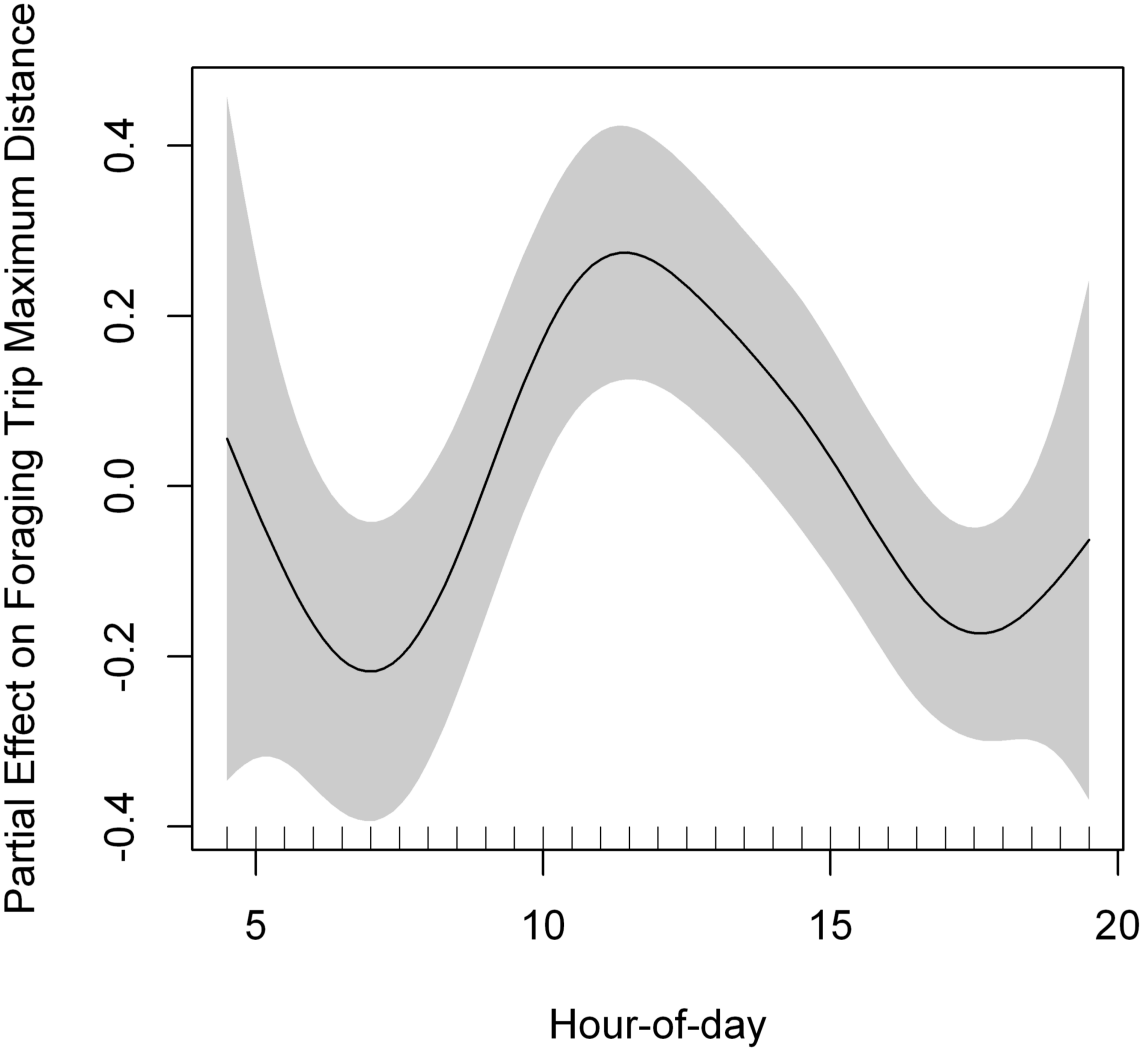
Partial effect of hour-of-day in the model fitted to lesser kestrel foraging trip maximum distance from the colony. A penalized smoothing spline of 4.70 degrees of freedom was adjusted to hour-of-day. Grey shading represents the standard error of the mean effect. Sample size = 444 foraging trips.

**Fig 5:**
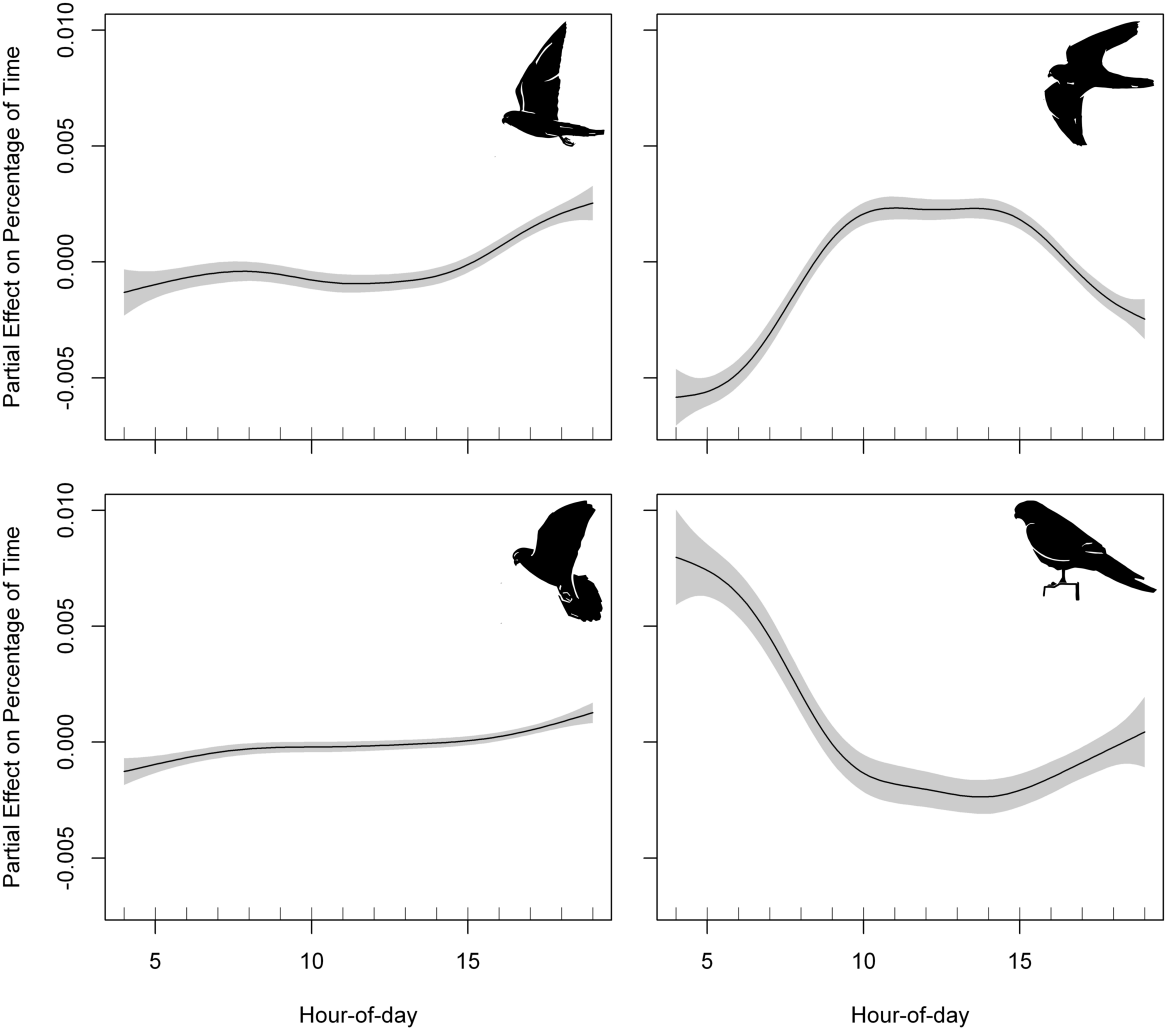
Partial effect of departure time (hour-of-day) on the percentage of time allocated to each behavior along foraging trips. Flapping flight (upper left panel), soaring-gliding flight (upper right panel), hovering flight (bottom left panel) and perching (bottom right panel). Penalized smoothing splines of 4.78, 7.00, 3.82 and 5.55 degrees of freedom were adjusted to hour-of-day for flapping flight, soaring-gliding flight, hovering flight, and perching, respectively. Grey shading represents the standard error of the mean effect. Sample size = 444 foraging trips.

We recorded 444 foraging trips, a mean of 74 ± 83.15 foraging trips per individual. The best GAMM fitted to foraging trip duration included phenological period and hour-of-day as predictors (Table 6, S3 Table). Lesser kestrels reduced foraging trip duration as the day progressed (Fig 3). The best GAMM fitted to foraging trip maximum distance included sex and departure time (hour-of-day) as predictors (Table 6, S3 Table). Individuals went farther from the colony during foraging trips departing at noon, and made shorter flights in the morning and in the evening (Fig 4). In contrast, foraging trip ODBA (energy expenditure) was not affected by hour-of-day, indicating that kestrels spent a similar amount of energy per foraging trip throughout the day (S4 Table). Lesser kestrels allocated on average more than 82% of foraging trip time and more than 96% of foraging trip energy (ODBA) to flight behaviors (Table 7). The best GAMM fitted to all variables of time and energy expenditure per behavior at the foraging trip level included hour-of-day as predictor (Table 6, S5 Table, S3 Fig). Time allocation to flapping and hovering flights per foraging trip tended to remain more or less constant as the day progressed, both increasing in the afternoon (Fig 5). Time devoted to soaring-gliding flights per foraging trip showed a positive curvilinear response to hour-of-day, reaching the maximum at noon (Fig 5). Time allocated to perching behavior per foraging trip decreased as the day progressed, showing a minimum at noon and increased again in the afternoon (Fig 5).

**Table 6:** Evaluation of the relative importance of each predictor in the GAMMs fitted to response variables analyzed at the foraging trip level. ΔAIC indicates the difference between the best model and the same model adding (negative values) or removing (positive values) the target predictor (depending on the predictors included in the best model). The higher the ΔAIC, the higher the relative importance of the predictor in the model. The predictors are coded as follows: Phenological Period = “PP”, sex = “S”, and hour-of-day = “H”. Hour-of-day was smoothed with a spline. Sample size = 444 foraging trips.

Response Variables		Best Model ΔAIC = 0	PP ΔAIC	S ΔAIC	H ΔAIC
Duration		H + PP	17.95	- 1.47 (Second best model)	25.39
Maximum Distance		H + S	- 0.48 (Second best model)	5.15	32.59
Time allocation to	Flapping	H	- 4.38	- 14.36	92.21
	Soaring-Gliding	H	- 12.93	- 12.85	379.08
	Hovering	H	- 16.02	- 14.65	47.68
	Perching	H	- 3.11	- 12.78	174.87

**Table 7:** Percentage of time and energy (ODBA) expenditure in different behaviors (mean value ± standard deviation) during the entire foraging trip, commuting flights and foraging event of the lesser kestrel. Sample size = 444 foraging trips, 888 commuting flights and 444 foraging events.

Level	Behavior	Time Investment (%)	ODBA Investment (%)
Foraging Trip	All (Commuting flights)	33.00 ± 29.94	33.64 ± 28.74
	All (Foraging event)	67.00 ± 29.94	66.36 ± 28.74
	Flapping	31.84 ± 15.58	55.85 ± 14.19
	Soaring-Gliding	43.01 ± 22.33	27.98 ± 16.22
	Hovering	7.33 ± 5.39	12.39 ± 6.26
	Perching	17.82 ± 24.82	3.78 ± 6.47
Commuting Flights	Flapping	40.78 ± 24.99	68.53 ± 22.76
	Soaring-Gliding	53.56 ± 25.39	30.44 ± 18.44
Foraging Event	Hovering	15.87 ± 17.18	26.82 ± 19.95
	Perching	26.22 ± 33.47	5.56 ± 8.72

### Segment level

We recorded 888 commuting flights (outwards and inwards) and 444 foraging events in foraging trips.

**Fig 6:**
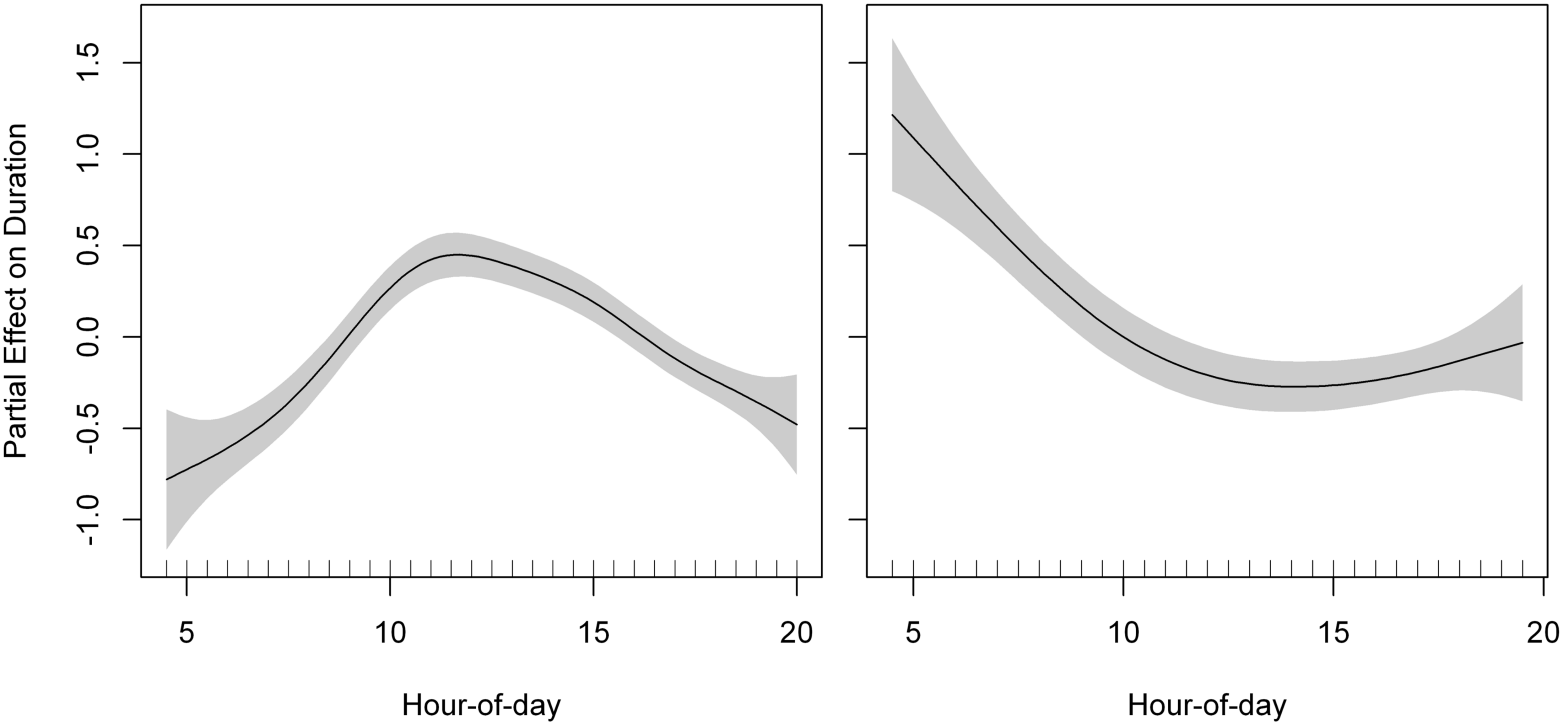
Partial effect of departure time (hour-of-day) on duration of commuting flights (left panel) and duration of foraging events (right panel). Penalized smoothing splines of 4.89 and 2.84 degrees of freedom were adjusted to hour-of-day for commuting flight and foraging event duration, respectively. Grey shading represents the standard error of the mean effect. Sample size = 888 commuting flights and 444 foraging events.

**Fig 7:**
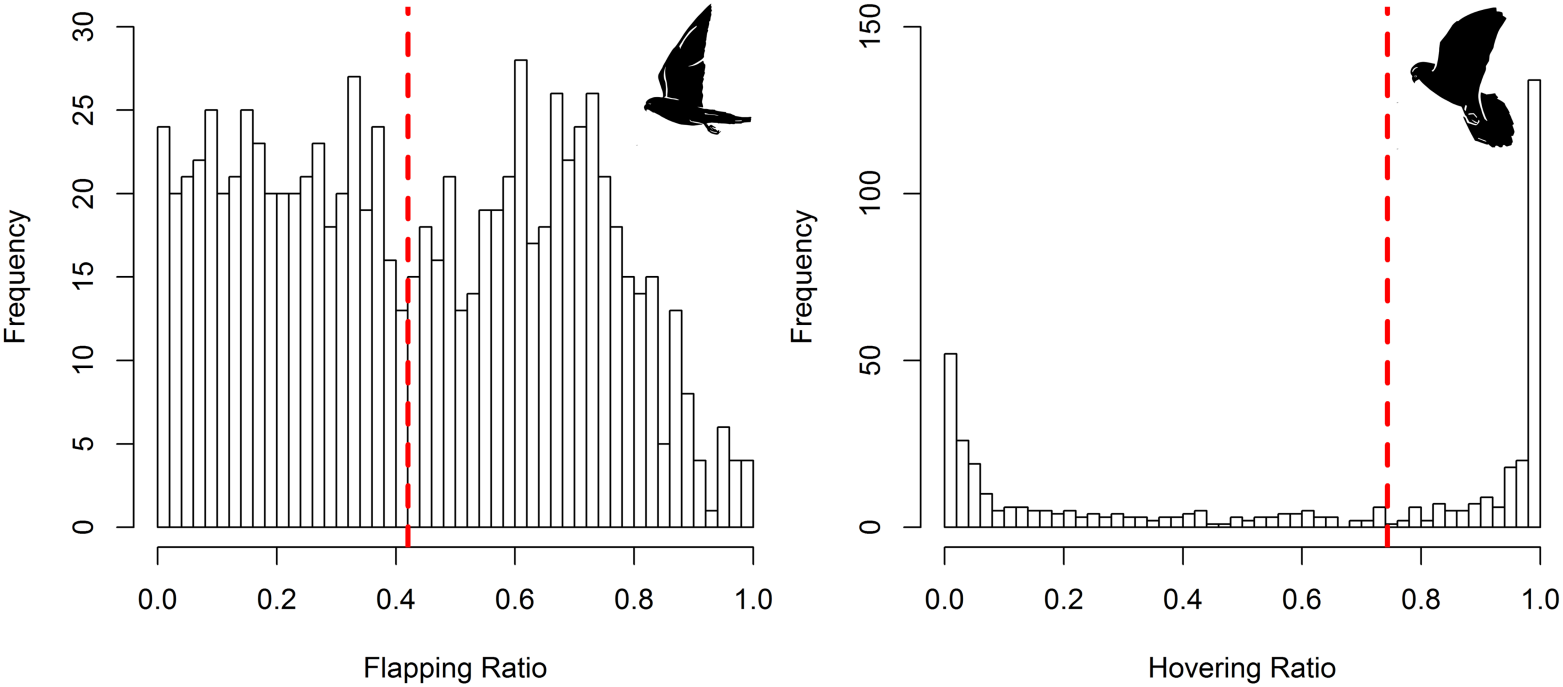
Frequency histogram of flapping (left panel) and hovering ratio (right panel) during commuting flights and foraging events, respectively, of foraging trips. The red dashed lines indicate the median value of ratios. Sample size = 888 commuting flights and 444 foraging events.

**Fig 8:**
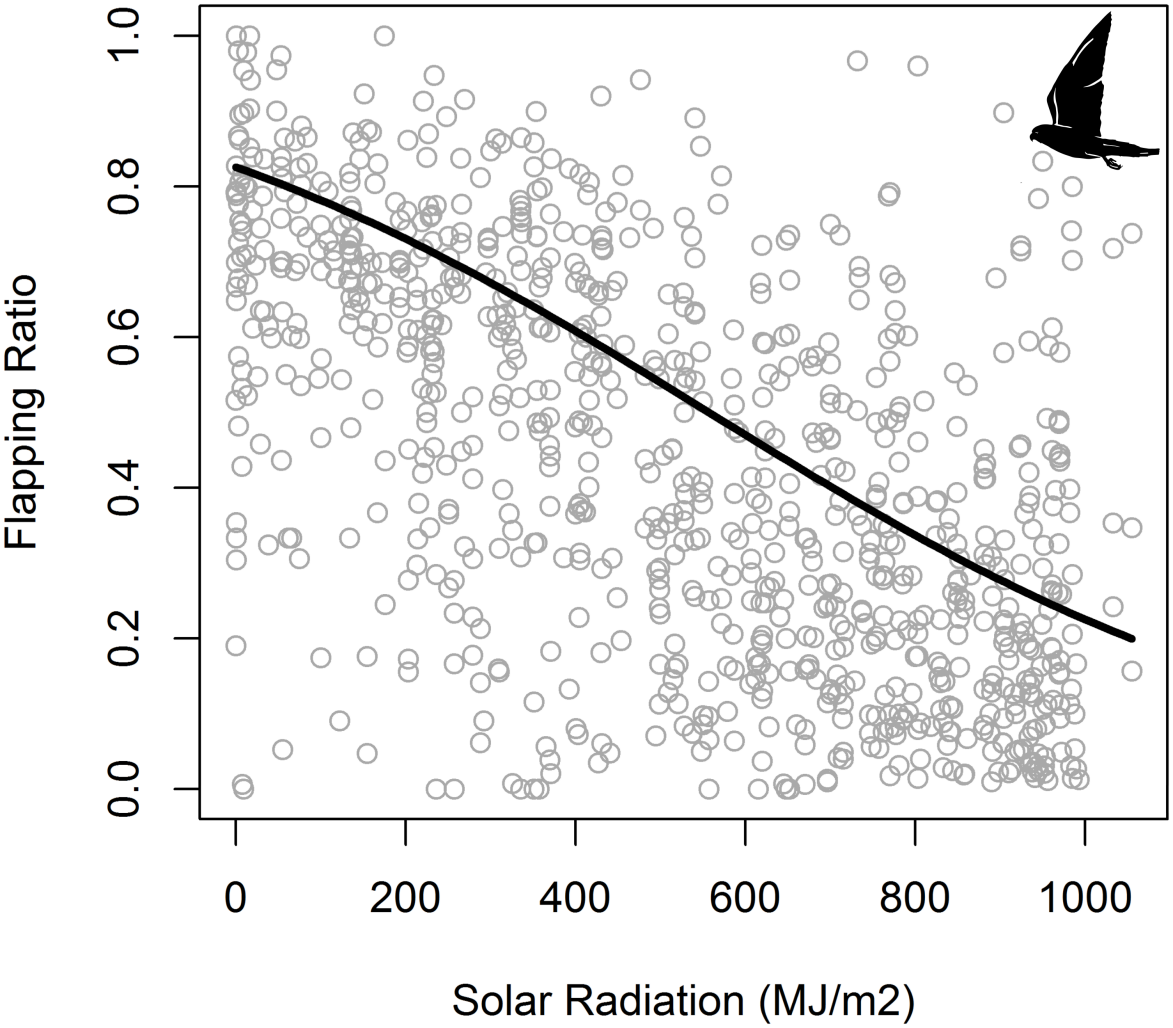
Effect of solar radiation on flapping ratio of lesser kestrel commuting flights predicted by the GLMM. Circles represent the observed flapping ratio of commuting flights and the solid line represents the model prediction. Sample size = 888 commuting flights.

For commuting flight duration, the best GAMM included commuting flight type, and hour-of-day as predictors (Table 8). For commuting flight ODBA, the best GAMM included sex, commuting flight type, and hour-of-day as predictors (Table 8). Outward flights were shorter and had lower energy expenditure (ODBA) than inward flights (S3 Table). Kestrels increased commuting flight duration and energy spent (ODBA) as the day progressed, reaching a maximum at noon and decreasing again towards the sunset (Fig 6, S3 Table, S4 Fig). We obtained on average a flapping ratio of 0.43 ± 0.26 that indicated a slight dominance of soaring-gliding over flapping during commuting flights (Fig 7). Flapping ratio showed a negative linear response to solar radiation (Fig 8). Kestrels tended to flap their wings more frequently during inwards that during outward flights (Table 9).

**Table 8:** Evaluation of the relative importance of each predictor in the GAMMs fitted to response variables analyzed at foraging trip segment level. ΔAIC indicates the difference between the best model and the same model adding (negative values) or removing (positive values) the target predictor (depending on the predictors included in the best model). The higher the ΔAIC, the higher the relative importance of the predictor in the model. The predictors are coded as follows: Phenological Period = “PP”, sex = “S”, hour-of-day = “H”, commuting flight type = “CF”, air temperature = “T” and wind speed = “W”.—indicates predictor not considered in the model. Predictors hour-of-day, air temperature, and wind speed were smoothed with splines. Sample size = 888 commuting flights and 444 foraging events.

Level	Response Variables	Best Model ΔAIC = 0	PP ΔAIC	S ΔAIC	H ΔAIC	CF ΔAIC	T ΔAIC	W ΔAIC
Commuting Flights	Duration	H + CF	- 8.76	1.32 (Second best model)	123.21	30.52	-	-
	ODBA	H + S + CF	- 4.20	10.13	29.48	78.21	-	-
Foraging Events	Duration	H + PP	31.94	1.71 (Second best model)	118.91	-	-	-
	Hovering Ratio	T + PP + W	6.48	0.56 (Second best model)	-	-	4.59	18.29

**Table 9:** Estimates (β), standard error (S.E.) and statistical significance of predictors included in the GLMM fitted to flapping ratio of lesser kestrel commuting flights. Statistically significant predictors are shown in bold. Sample size = 888 commuting flights.

Predictors	β	S.E.	χ^2^	p-value
Intercept	0.83	0.57	-	-
**Solar Radiation**	- 0.0004	0.50	269.26	< 0.001
Wind Speed	- 0.001	0.51	0.02	0.89
**Commuting Flight Type** (Inwards)	0.05	0.52	20.44	< 0.001
Sex (Female)	- 0.02	0.58	0.39	0.53
**Phenological Period** (Incubation)	0.07	0.56	5.04	0.02

**Fig 9:**
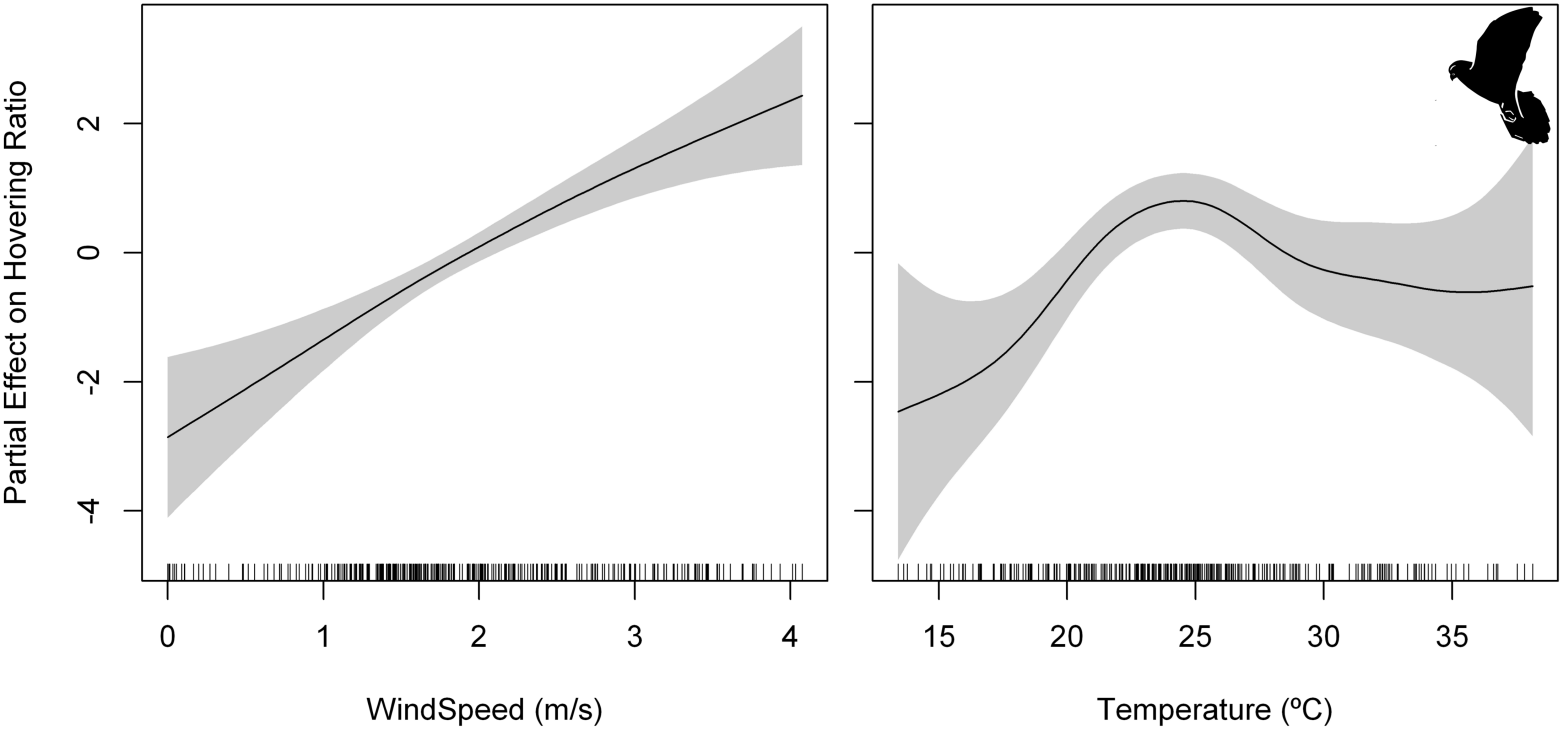
Partial effect of wind speed (left panel) and air temperature (right panel) on the model fitted to hovering ratio of lesser kestrel foraging events. Penalized smoothing splines of 1.54 and 4.15 degrees of freedom were adjusted to wind speed and air temperature, respectively. Grey shading represents the standard error of the mean effect. Sample size = 444 foraging events.

For foraging event duration, the best GAMM included phenological period and hour-of-day as predictors (Table 8, S3 Table). Foraging event duration decreased as the day progressed reaching a minimum at noon and increased slightly towards the sunset (Fig 6). We did not find any statistically significant effect of hour-of-day on foraging event energy expenditure (ODBA) (S6 Table). We obtained on average a hovering ratio of 0.58 ± 0.41 that indicated a relative dominance of hovering flights over perching behavior during the foraging events (Fig 7). For hovering ratio, the best GAMM included phenological period, wind speed, and air temperature as predictors (Table 8, S3 Table). Hovering ratio increased linearly with wind speed, and also with increasing air temperature, reaching a threshold at 25°C above which hovering ratio showed a stable or slightly decreasing trend (Fig 9).

### Hovering and perching bouts

**Fig 10:**
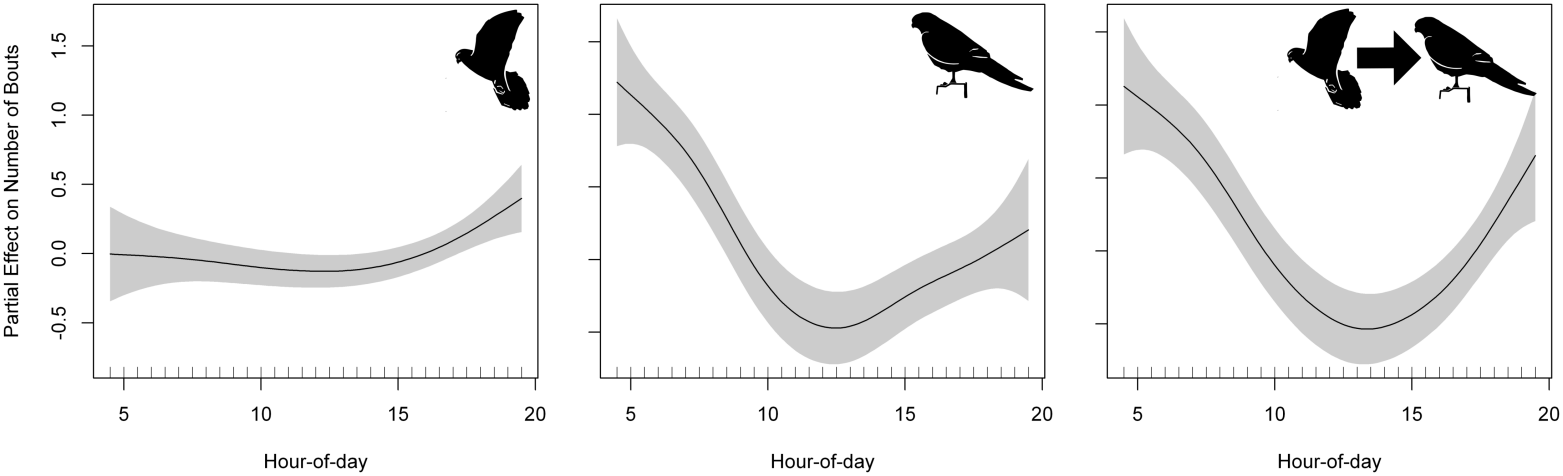
Partial effect of hour-of-day in the models fitted to the number of hovering bouts (left panel), the number of perching bouts (middle panel) and the number of hovering-perching bouts (right panel) per foraging event. Penalized smoothing splines of 2.35, 3.56 and 3.47 degrees of freedom were adjusted to hour-of-day for the number of hovering bouts, perching bouts, and hovering-perching bouts per foraging event, respectively. Grey shading represents the standard error of the mean effect. Sample size = 444 foraging events.

We identified 4,933 hovering bouts (a mean of 8.91 ± 11.60 bouts/foraging event) and 2,798 perching bouts (a mean of 4.65 ± 10.04 bouts/foraging event). For the number of hovering bouts per foraging event, the best GAMM included phenological period and hour-of-day (Table 10, S3 Table). Similarly, for the number of perching bouts per foraging event, the best model included also phenological period and hour-of-day. The number of hovering bouts per foraging event remained constant along the day but it showed an increase towards the sunset. The number of perching bouts per foraging event decreased as the day progressed reaching a minimum at noon and then increased again towards the sunset (Fig 10).

**Table 10:** Evaluation of the relative importance of each predictor in the GAMMs fitted to the number of hovering bouts, perching bouts and hovering-perching bouts per foraging event. ΔAIC indicates the difference between the best model and the same model adding (negative values) or removing (positive values) the target predictor (depending on the predictors included in the best model). The higher the ΔAIC, the higher the importance of the predictor in the model. The predictors are coded as follows: Phenological Period = “PP”, individual sex = “S”, and hour-of-day = “H”. Hour-of-day was smoothed with a spline. Sample size = 444 foraging events.

Response Variables	Best Model ΔAIC = 0	PP ΔAIC	S ΔAIC	H ΔAIC
# Hovering Bouts	H + PP	8.09	- 2.04	8.78
# Perching Bouts	H + PP	52.18	- 3.51	98.90
# Hovering-Perching Bouts	H + PP	18.87	- 2.98	96.91

We identified 476 hovering-perching bouts (hovering followed by perching—a proxy for self-feeding activity—, a mean of 1.07 ± 2.10 bouts/foraging event). For the number of hovering-perching bouts per foraging event, the best GAMM included also phenological period and hour-of-day as predictors (Table 10, S3 Table). The number of hovering-perching bouts per foraging event decreased as the day progressed reaching a minimum at noon and then increased again towards the sunset (Fig 10). The number of hovering-perching bouts per foraging event was higher in those foraging trips that end at a roost site (4.81 ± 4.17 hovering-perching bouts/event; Mann-Whitney U test, z = 6.91, p < 0.001, n = 16) versus those that end at the colony (0.93 ± 1.86 hovering-perching bouts/event, n = 428).

## Discussion

In this study, we were able to identify five different behaviors (flapping, soaring-gliding, hovering, perching and incubating/brooding) of free-ranging lesser kestrels during the breeding season through a combination of tri-axial accelerometry and GPS tracking. We obtained a behavior classification model that allowed us to classify kestrel behaviors from accelerometer data with high accuracy (Table 3). The use of tri-axial accelerometers and the behavior classification model allowed us to estimate the average energy expenditure of each lesser kestrel flight and hunting behavior using ODBA as a proxy (Table 2). Flight behaviors (flapping, soaring-gliding and hovering flights) require more energy than stationary behaviors (perching and incubating/brooding). Within the flight behaviors, flapping and hovering require three times more energy than soaring-gliding, as predicted by flight theory [57] and in agreement with empirical studies on bird flight dynamics [48,58]. Therefore, accelerometers provided us with an efficient tool to study how lesser kestrels partition their time and energy into different behaviors throughout the day.

We observed sexual differences in the daily energy and time activity budget of the lesser kestrel (Table 4). Males spend more time and energy in flight behaviors and less time and energy in stationary behaviors than females on a daily basis (Table 5, S2 Table). This different daily level of activity between sexes is in agreement with the role specialization in raptors: males usually provision their mate and/or offspring whereas females incubate, brood the chicks, and defend the nest [52,59]. The higher flight activity of the male is consistent with its role as prey provider at the nest, previously reported in this species. In a similar way, the higher allocation of females to stationary behaviors is consistent with the elevated daily nest attendance described for this species [60]. Contrary to the general trend in raptors, in the lesser kestrel both sexes share the incubation of eggs [60], explaining the decline in energy and time allocation to flight behaviors and the higher proportion of incubating/brooding behavior during the incubation period versus the nestling period.

**Fig 11:**
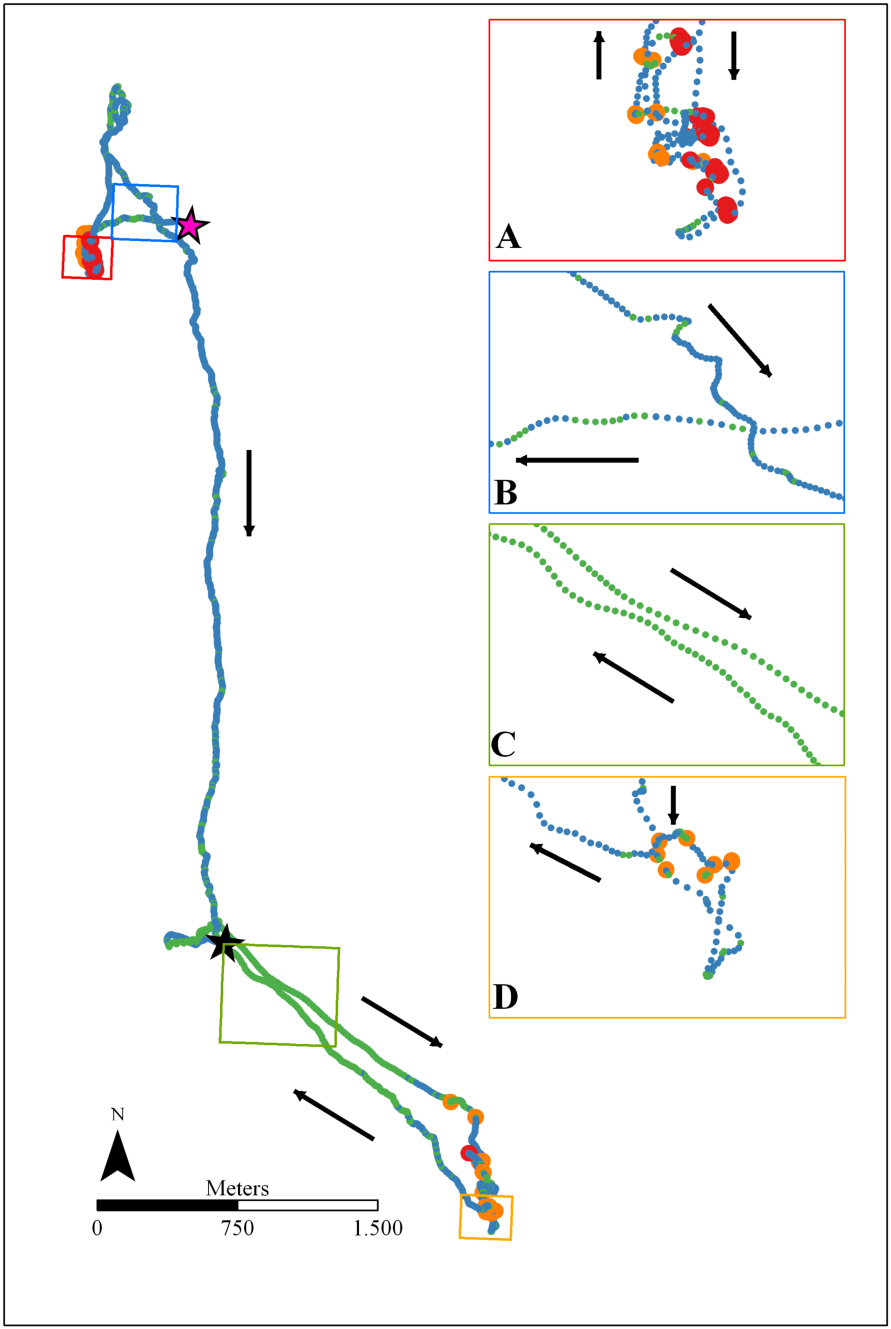
Example of two foraging flights with different strategies with behaviors tagged using the final classification model. GPS fixes recorded at 1-second frequency. The colors of the icons represent different behaviors: flapping (blue), soaring-gliding (green), hovering (orange), and perching (red). The black star indicates the breeding colony and the pink star indicates an overnight roost. Black arrows indicate movement direction. Boxes include a zoomed view of the foraging trip segment indicated with the same color in the main panel. Early morning hunting behavior: A) foraging event (hovering ratio = 0.02); (B) commuting flights (mean flapping ratio = 0.71). Noon hunting behavior: (C) commuting flights (mean flapping ratio = 0.29); and (D) foraging event (hovering ratio = 0.77).

The lesser kestrel shows a non-uniform daily distribution of behaviors. Individual kestrels spend the nighttime resting or incubating/brooding (stationary behaviors) in accordance with being a diurnal species. Kestrels dedicate the daytime mostly to flight behaviors, that is, individuals allocate almost the complete daylight period to foraging activities, especially during the nestling period. Our results indicate a dramatic change with time of the day on the percentage of time and energy devoted to different behaviors along the foraging trips. This suggests that kestrels show a flexible foraging strategy throughout the diurnal cycle (Fig 11). During the commuting flights, lesser kestrels can either decide to fly using either flapping or soaring-gliding flights and such decision follows a circadian pattern governed by solar radiation. Kestrels use flapping flights to commute between the colony and the foraging areas early in the morning. As the day progresses, soaring-gliding becomes the predominant strategy reaching the maximum around midday, and then the use of flapping flights increases again as the sunset approaches (Fig 5). When the flapping ratio is analyzed, the intensity of solar radiation arises as the most important weather variable in determining the behavioral decision about which flight strategy to use during commuting flights (Table 9). Solar radiation is the causal agent for thermal formation since thermal currents result from the differential heating of the ground and the low level of the atmosphere by the sun [61], so it can be taken as a proxy for thermal development. Thus, as solar radiation increases the flapping ratio decreases meaning that kestrels progressively replace flapping with soaring-gliding flights as thermals get stronger. This quantitative result supports the qualitative ones previously obtained for this species when comparing foraging trips with and without thermal soaring events, which were identified on high-frequency GPS tracks [38]. This allows us to confirm that as solar radiation increases, kestrels harvest more kinetic energy from the atmosphere, transform it into potential energy by circling up in stronger thermals and fly by gliding with lower energy cost (as estimated by ODBA). Individual kestrels take advantage of this reduction in flight cost to fly farther from the colony during foraging trips around midday when solar radiation is stronger and consequently using a higher percentage of soaring-gliding flights (Fig 4). This, together with the lower cross-country speed of thermal soaring, results in longer (Fig 6) and more costly (S4 Fig) commuting flights at the central hours of the day. We also found that kestrels used a higher proportion of flapping flights during the inward flights in comparison to the outward flights (Table 9). Kestrels carrying a single prey item to the colony during inward flights carries an extra-load that implies an increase in the sinking rate of the individual, that is, an increase in its downward speed in relation to the forward speed when gliding [57]. Consequently, kestrels probably use more flapping flights when returning to the colony in order to compensate the extra weight. This would explain the observed significant higher energy expenditure of inward flights in comparison to outward flights (S3 Table). Wind speed did not influence the flapping ratio of commuting flights, in agreement with the absence of a strong effect of wind on lesser kestrel flights previously reported at our study site [39].

During the foraging events of the foraging trips, kestrels can decide either to hunt by using hovering flights (i.e. active hunting) or perch-hunting (i.e. sit-and-wait strategy) and such decision is also influenced by the time of the day. Kestrels mostly hunt from perches early in the morning, but as the day progresses they switch to hovering flights that remain dominant until close to sunset when perch-hunting increases again (Fig 5). When the hovering ratio is analyzed, wind speed and air temperature arise as important weather variables in determining the behavioral decision of which hunting strategy to use during foraging events (Table 8). As wind speed increases the hovering ratio also increases, indicating that kestrels gradually replace the perch-hunting with the hovering flights as wind intensifies (Fig 9). This is in agreement with what was found in the American kestrel (*Falco sparverius*), a closely related species, that increases the percentage of time devoted to hovering flights as wind speed increases until a threshold above which that percentage decreases [51]. Vlachos *et al*. [62] found a negative linear relationship between wind speed and hovering hunting rate in the lesser kestrel. In our study area, maximum wind speed is probably too low (~ 4 m/s, see [39]) during kestrel breeding season to cause any negative response in the hovering ratio. Hovering flights are an energetically costly behavior, 24 times more energy per time unit than perching (according to ODBA, Table 2). However, kestrels could take advantage of the lift force originated by winds that would help them to remain aloft with less reliance on wing beats during hovering flights [63]. Therefore, as wind speed increases along the day, kestrels would experience stronger lifts and consequently would reduce energy expenditure of hovering flights to a greater extent. Nevertheless, the bimodal frequency distribution of the hovering ratio (Fig 7) seems to not be completely explained by a gradual switch from perch-hunting to hovering flight mediated by wind speed. Here, the effect of air temperature on the hovering ratio would play an important role. As air temperature increases the hovering ratio also increases, that is, kestrels change from the sit-and-wait hunting strategy to the active hunting strategy as the day gets warmer until approximately 25°C, above which the hovering ratio stabilizes (Fig 9). We hypothesize that this change in hunting strategy of the lesser kestrel may be mediated by the activity pattern of its preferred prey. The diet of the lesser kestrel changes through the breeding season, but it is predominantly composed by bush crickets (family Tettigoniidae, mostly genus *Ephippiger* and *Decticus*) during the incubation and nestling periods in our study area [64]. Bush crickets show marked stridulatory and locomotory activities that are highly determined by air temperature [65,66]. *Ephippiger ephippiger* males sing to attract mate and, in response, females in synchrony increase their mobility when air temperature increases above 17°C [67]. Therefore, early in the morning when air temperature is low, preferred prey would be less active and subsequently more difficult to be found by kestrels, so individuals adopt a sit-and-wait hunting strategy to save energy costs, although it takes longer to detect and capture a prey. As the day progresses and gets warmer, bush crickets become more active and consequently are easier to be detected by kestrels, triggering a change of individual strategy to active hunting, which requires more energy per time unit but requires less time to encounter prey (as can be seen in our data of foraging event duration). However, the hovering-specific energy expenditure would decline as wind speed increases along the day, in this way relaxing the trade-off between the two hunting strategies. It is not surprising that kestrels modify their foraging strategy in response to prey availability since food abundance and density have been identified as key factors affecting foraging behavior in numerous species [25,68–72]. Thus, the combination of wind conditions, prey activity and hunting strategy efficiency results in the observed daily pattern of foraging event duration: foraging events are longer early in the morning but its duration decreases as the day progresses (following an increase in bush cricket activity) (Fig 6). Although the two hunting strategies employed have dramatically different costs per time unit, the energy expenditure associated with foraging events did not change along the day (S6 Table). Consequently, as the energy expenditure of the foraging event is the major part of the overall energy expenditure of the foraging trip (Table 7), this neither changes along the day (S4 Table).

Hovering flights constitute the main hunting strategy of kestrels [34], but it is also a recurring strategy to search for food among insects, bats, or hummingbirds [73–75], so its identification should be key when studying foraging ecology. Tri-axial acceleration signature of both flapping and soaring-gliding flights has been described on numerous bird species [18,47,48,76], but this is, to our knowledge, the first time that the acceleration signature of hovering flights is described. A limitation of our study is that we were not able to distinguish whether kestrels were successful or not in capturing prey after performing a hovering or perching bout. Nevertheless, we can infer the number of prey captured per foraging trip because the foraging behavior of the lesser kestrel has been extensively studied in the field. In the lesser kestrel, the number of hovering bouts necessary to make a strike has been estimated at 4.3–5.5 that are successful on average 39–73% of the times [62,77–79]. Zank and Kemp [79] recorded a 59% success rate of lesser kestrel when hunting from perches, which is similar to the 54% recorded in the American kestrel [80]. We obtained a mean of 8.91 hovering bouts and a mean of 4.65 perching bouts per foraging event. Therefore, being conservative, individual kestrels would be capturing on average a single prey per foraging event, which is what would be expected as they have to return to the colony to feed their offspring and they only carry one item at a time. Another limitation of our study is the difficulty of identifying when kestrels capture prey to be self-consumed. Observations in the field support that kestrels often fly to a perch to eat the prey after its capture [51], so we can consider the number of hovering-perching bouts as an indicator of kestrel self-feeding activity. The higher number of hovering-perching bouts observed in those foraging trips that end in overnight roost sites, instead of returning to the colony, supports this assumption. The number of hovering-perching bouts shows a marked daily pattern: the rate is high early in the morning, decreases as the day progresses and slightly increases towards the sunset (Fig 10). Therefore, breeding kestrels could feed themselves especially during the first hours after sunrise and also close to sunset. In addition, early in the morning, when bush crickets are supposed to be less active, lesser kestrels might forage using perch hunting on any prey they could find, mainly beetles, small grasshoppers, and crickets, that also appear in the lesser kestrel diet but are smaller and less energetically rewarding than bush crickets [64]. At this time of the daylight period there are no thermals so the cost of returning to the colony by using flapping flights would outweigh the benefits provided by the prey captured, so it is more efficient to use them for self-feeding instead of provisioning the offspring, as found in other species of genus *Falco* [51,81]. Close to sunset, lesser kestrels would find a similar scenario in relation to thermals, but they hover more often than during the morning. The reason may be because wind speed is higher during the evening so kestrels can hover with lower energy cost than in the morning. Furthermore, as the day progresses towards the sunset, the difference between air and ground temperatures reduces and consequently thermal updraft strength weakens, although air temperature is still high and bush crickets are active [82]. This would allow lesser kestrel breeders to continue capturing their preferred prey by using hovering flights with a similar success rates to those obtained around midday, but in this case to feed themselves (because the lack of thermals would increase the cost of an inward flight to the colony). It is common that the last foraging trip of the day ends at an overnight roost site, especially for kestrel males. By staying in these roosts, individuals save the flight costs of returning to the colony using flapping flights at the end of the daylight period. Moreover, individuals also save the flight cost associated with the outward commuting flight the next morning, since they are already within a suitable foraging patch located far from the colony. This is supported by the large variability in foraging trip maximum distance from the colony that we find early in the morning and late in the evening (Fig 4). Therefore, kestrels seem to time their self-feeding activity to those periods of the day when commuting flight costs outweigh the potential benefits of prey transport to the nest.

## Conclusions

GPS and tri-axial accelerometer data allowed us to classify lesser kestrel behavior successfully, supporting the efficiency of this methodology to study behaviors in free-ranging birds. Our results indicate that the role specialization of the lesser kestrel explains the differences between sexes in daily energy expenditure during the breeding season. They also show that lesser kestrel behavioral decisions about which flight and hunting strategies to use during foraging trips are influenced by environmental conditions (solar radiation, wind speed and air temperature) that change throughout the day resulting in marked circadian patterns of foraging strategy. Interestingly, although the two hunting strategies used by kestrels, hovering versus perch-hunting, differ dramatically in costs per time unit, the energy expended per foraging trip does not vary through the day, suggesting that kestrels have a quite fixed energy budget per foraging trip to which they adjust their flight and hunting strategies in response to the environmental conditions.

## Supporting information

S1 Fig Position of the tracking device on the lesser kestrel’s back and direction of the three axes in which acceleration was measured. (TIF)

S2 Fig Daily trend of solar radiation, air temperature and wind speed obtained by adjusting a smoothing spline with five degrees of freedom. Sample size = 1,056 weather data samples from 22 days (3^rd^– 24^th^ June).
(TIF)

S3 Fig Partial effect of hour-of-day in the model fitted to foraging trip energy expenditure per behavior. Flapping flight (upper left panel), soaring-gliding flight (upper right panel), hovering flight (bottom left panel) and perching (bottom right panel). Penalized smoothing splines of 6.29, 6.64, 4.67 and 4.20 degrees of freedom were adjusted to hour-of-day for flapping flight, soaring-gliding flight, hovering flight and perching, respectively. Grey shading represents the standard error of the mean effect. Sample size = 444 foraging trips.
(TIF)

S4 Fig Partial effect of hour-of-day in the model fitted to lesser kestrel commuting flight ODBA. A penalized smoothing spline of 3.34 degrees of freedom was adjusted to hour-of-day. Grey shading represents the standard error of the mean effect. Sample size = 888 commuting flights.
(TIF)

S1 Table Mean confusion matrix for the three classification model obtained from the jack-knife procedure. We built this matrix adding the results of the confusion matrix of each of the three models, which were built with the data of two of the three kestrels to classify behaviors of the third. Soaring-gliding and incubating/brooding are indicated as Gliding and Incubating, respectively. Observations correctly classified per behavior are shown in bold.
(DOCX)

S2 Table Estimates (β) and standard error (S.E.) of predictors included in the GLMM fitted to daily energy expenditure in different behaviors of the lesser kestrel. Statistically significant predictors are shown in bold: * p < 0.5, ** p < 0.01, *** p < 0.001. Sample size = 35 complete days of tracking.
(DOCX)

S3 Table Estimate (β) ± standard error (S.E.) predicted for sex, phenological period, and commuting flight type included in the GAMMs fitted to variables analyzed at the foraging trip or segment levels. Predictors included in the best model fitted to each variable as response are shown in bold.–indicates predictor not considered in the model. Sample Size = 444 foraging trips, 888 commuting flights, and 444 foraging events.
(DOCX)

S4 Table Estimates (β), standard error (S.E.) and statistical significance of predictors included in the GLMM fitted to foraging trip ODBA. Statistically significant predictors are shown in bold. Sample size = 444 foraging trips.
(DOCX)

S5 Table Evaluation of the importance of each predictor separately in the GAMMs fitted to response variables of lesser kestrel foraging trips. ΔAIC indicated the difference between the best model and the same model adding (negative values) or removing (positive values) the target predictor (depending on the predictors included in the best model). The higher the ΔAIC, the higher the importance of the predictor in the model fit. The predictors are classed as follows: Phenological Period as “PP”, individual sex as “S”, and hour-of-day as “H”. Hour-of-day was smoothed with a spline. Sample size = 444 foraging trips.
(DOCX)

S6 Table Estimates (β), standard error (S.E.) and statistical significance of predictors included in the GLMM fitted to foraging event ODBA. Statistically significant predictors are shown in bold. Sample size = 444 foraging trips.
(DOCX)

## References

[1] Rodríguez A, Negro JJ, Mulero M, Rodríguez C, Hernández-Pliego J, Bustamante J. The eye in the sky: Combined use of unmanned aerial systems and GPS data loggers for ecological research and conservation of small birds. PLoS One. 2012;7 e50336 10.1371/journal.pone.0050336 23239979 PMC3519840

[2] Wilmers CC, Nickel B, Bryce CM, Smith JA, Wheat RE, Yovovich V, et al The golden age of bio-logging: How animal-borne sensors are advancing the frontiers of ecology. Ecology 2015;96: 1741–1753. 26378296 10.1890/14-1401.1

[3] Nathan R, Getz WM, Revilla E, Holyoak M, Kadmon R, Saltz D, et al A movement ecology paradigm for unifying organismal movement research. Proc Natl Acad Sci. 2008;105: 19052–19059. 10.1073/pnas.0800375105 19060196 PMC2614714

[4] Watanabe S, Izawa M, Kato A, Ropert-Coudert Y, Naito Y. A new technique for monitoring the detailed behaviour of terrestrial animals: A case study with the domestic cat. Appl Anim Behav Sci. 2005;94: 117–131.

[5] Graf PM, Wilson RP, Qasem L, Hackländer K, Rosell F. The use of acceleration to code for animal behaviours: A case study in free-ranging Eurasian beavers *Castor fiber*. PLoS One. 2015;10: e0136751 10.1371/journal.pone.0136751 26317623 PMC4552556

[6] Shepard E, Wilson R, Quintana F, Gómez Laich A, Liebsch N, Albareda DA, et al Identification of animal movement patterns using tri-axial accelerometry. Endanger Species Res. 2008;10: 47–60.

[7] Chimienti M, Cornulier T, Owen E, Bolton M, Davies IM, Travis JMJ, et al The use of an unsupervised learning approach for characterizing latent behaviors in accelerometer data. Ecol Evol. 2016;6: 727–741. 10.1002/ece3.1914 26865961 PMC4739568

[8] Cooke SJ, Hinch SG, Wikelski M, Andrews RD, Kuchel LJ, Wolcott TG, et al Biotelemetry: A mechanistic approach to ecology. Trends Ecol Evol. 2004;19: 334–43. 10.1016/j.tree.2004.04.003 16701280

[9] Brown DD, Kays R, Wikelski M, Wilson R, Klimley AP. Observing the unwatchable through acceleration logging of animal behavior. Anim Biotelemetry. 2013;1: 20.

[10] Elliott KH, Le Vaillant M, Kato A, Speakman JR, Ropert-Coudert Y. Accelerometry predicts daily energy expenditure in a bird with high activity levels. Biol Lett. 2013;9 10.1098/rsbl.2012.0919 23256182 PMC3565507

[11] Halsey LG, Shepard ELC, Hulston CJ, Venables MC, White CR, Jeukendrup AE, et al Acceleration versus heart rate for estimating energy expenditure and speed during locomotion in animals: tests with an easy model species, *Homo sapiens*. Zoology. 2008;111: 231–241. 10.1016/j.zool.2007.07.011 18375107

[12] Wilson RP, White CR, Quintana F, Halsey LG, Liebsch N, Martin GR, et al Moving towards acceleration for estimates of activity-specific metabolic rate in free-living animals : The case of the cormorant. J Anim Ecol. 2006;75: 1081–1090. 10.1111/j.1365-2656.2006.01127.x 16922843

[13] Green JA, Halsey LG, Wilson RP, Frappell PB. Estimating energy expenditure of animals using the accelerometry technique: Activity, inactivity and comparison with the heart-rate technique. J Exp Biol. 2008;212: 471–482. 10.1242/jeb.026377 19181894

[14] Halsey LG, Shepard ELC, Quintana F, Gomez Laich A, Green JA, Wilson RP. The relationship between oxygen consumption and body acceleration in a range of species. Comp Biochem Physiol Part A. 2009;152: 197–202. 10.1016/j.cbpa.2008.09.021 18854225

[15] Gleiss AC, Dale JJ, Holland KN, Wilson RP. Accelerating estimates of activity-specific metabolic rate in fishes: Testing the applicability of acceleration data-loggers. J Exp Mar Bio Ecol. 2010;385: 85–91.

[16] Brown DD, Montgomery RA, Millspaugh JJ, Jansen PA, Kays R. Selection and spatial arrangement of rest sites within northern tamandua home ranges. J Zool. 2014;293: 160–170.

[17] Tanida H, Koba Y, Rushen J, de Passilé AM. Use of three-dimensional acceleration sensing to assess dairy cow gait and the effects of hoof trimming. Anim Sci J. 2011;82: 792–800. 10.1111/j.1740-0929.2011.00903.x 22111637

[18] Ropert-Coudert Y, Grémillet D, Kato A, Ryan PG, Naito Y, Le Maho Y. A fine-scale time budget of Cape gannets provides insights into the foraging strategies of coastal seabirds. Anim Behav. 2004;67: 985–992.

[19] Wilson RP, Griffiths IW, Mills MGL, Carbone C, Wilson JW, Scantlebury DM. Mass enhances speed but diminishes turn capacity in terrestrial pursuit predators. Elife. 2015;4: 1–18. 10.7554/eLife.06487 PMC4542338 26252515

[20] Whitney NM, Papastamatiou YP, Holland KN, Lowe CG. Use of an acceleration data logger to measure diel activity patterns in captive whitetip reef sharks, *Triaenodon obesus*. Aquat Living Resour. 2008;20: 299–305.

[21] Davis RW, Fuiman LA, Williams TM, Collier SO, Hagey WP, Kanatous SB, et al Hunting behavior of a marine mammal beneath the Antarctic fast ice. Science 1999;283: 993–996. 9974394 10.1126/science.283.5404.993

[22] Yoda K, Sato K, Niizuma Y, Kurita M, Bost C-A, Le Maho Y, et al Precise monitoring of porpoising behavior of Adélie penguins determined using acceleration data loggers. J Exp Biol. 1999;202: 3121–3126. 10539960 10.1242/jeb.202.22.3121

[23] MacArthur RH, Pianka ER. On optimal use of a patchy environment. Am Nat. 1966;100: 603–609.

[24] Saraux C, Robinson-Laverick SM, Le Maho Y, Ropert-Coudert Y, Chiaradia A. Plasticity in foraging strategies of inshore birds: How Little Penguins maintain body reserves while feeding offspring. Ecology 2011;92: 1909–1916. 22073782 10.1890/11-0407.1

[25] Chivers L, Lundy M, Colhoun K, Newton S, Houghton J, Reid N. Foraging trip time-activity budgets and reproductive success in the black-legged kittiwake. Mar Ecol Prog Ser. 2012;456: 269–277.

[26] Fossette S, Schofield G, Lilley MKS, Gleiss AC, Hays GC. Acceleration data reveal the energy management strategy of a marine ectotherm during reproduction. Funct Ecol. 2012;26: 324–333.

[27] Le Vaillant M, Wilson RP, Kato A, Saraux C, Hanuise N, Prud’homme O, et al King penguins adjust their diving behaviour with age. J Exp Biol. 2012;215: 3685–92. 10.1242/jeb.071175 23053365

[28] Weimerskirch H, Louzao M, De Grissac S, Delord K. Changes in wind pattern alter albatross distribution and life-history traits. Science 2012;335: 211–214. 10.1126/science.1210270 22246774

[29] Wakefield ED, Bodey TW, Bearhop S, Blackburn J, Colhoun K, Davies R, et al Space partitioning without territoriality in gannets. Science 2013;341: 68–70. 10.1126/science.1236077 23744776

[30] Orians GH, Pearson NE. On the theory of central place foraging In: Horn DH, Mitchell R, Stairs GR, editors. Analysis of ecological systems. Ohio State University Press, Columbus; 1979 pp. 155–177.

[31] Amélineau F, Péron C, Lescroël A, Authier M, Provost P, Grémillet D. Windscape and tortuosity shape the flight costs of northern gannets. J Exp Biol. 2014;217: 876–85. 10.1242/jeb.097915 24622894

[32] Spaar R. Flight strategies of migrating raptors: A comparative study of interspecific variation in flight characteristics. Ibis 1997;139: 523–535.

[33] Hedenström A. Migration by soaring or flapping flight in birds: The relative importance of energy cost and speed. Philos Trans R Soc Lond B Biol Sci. 1993;342: 353–361.

[34] Village A. The Kestrel. London: A&C Publishers Ltd, 36 Soho Square, London; 1990.

[35] Anderson RA, Karasov WH. Contrasts in energy intake and expenditure in sit-and-wait and widely foraging lizards. Oecologia 1981;49: 67–72. 10.1007/BF00376899 28309450

[36] Jaksic FM, Carothers JH. Ecological, morphological, and bioenergetic correlates of hunting mode in hawks and owls. Ornis Scand. 1985;16: 165–172.

[37] Aparicio JM. Actividad, selección del método de caza y balance energético diario de *Falco naumanni* durante el período premigratorio. Ardeola 1990;37: 163–178.

[38] Hernández-Pliego J, Rodríguez C, Bustamante J. Why do kestrels soar? PLoS One. 2015;10: e0145402 10.1371/journal.pone.0145402 26689780 PMC4687047

[39] Hernández-Pliego J, Rodríguez C, Bustamante J. Gone with the wind: Seasonal trends in foraging movement directions for a central place forager. Curr Zool. 2014;60: 604–615.

[40] Hernández-Pliego J, Rodríguez C, Bustamante J. A few long versus many short foraging trips: different foraging strategies of lesser kestrel sexes during breeding. Movement Ecology 2017; 5:8 10.1186/s40462-017-0100-6 28451434 PMC5404669

[41] Cramp S, Simmons KEL. The birds of the Western Palearctic. Oxford University Press, Oxford; 1980.

[42] Bustamante J. Predictive models for lesser kestrel *Falco naumanni* distribution, abundance and extinction in southern Spain. Biol Conserv. 1997;80: 153–160.

[43] IUCN. IUCN Red List of threatened species. Version 2013.2. 2013; Downloaded on 16/11/2013. www.iucnredlist.org

[44] Fernandez R, Martin A, Ortega F, Ales EE. Recent changes in landscape structure and function in a mediterranean region of SW Spain (1950–1984). Landsc Ecol. 1992;7: 3–18.

[45] Barron DG, Brawn JD, Weatherhead PJ. Meta-analysis of transmitter effects on avian behaviour and ecology. Methods Ecol Evol. 2010;1: 180–187.

[46] Hernández-Pliego J, Rodríguez C, Bustamante J. Data from: Why do kestrels soar? Movebank Data Repository; 2015 p. 10.5441/001/1.sj8t3r11 PMC4687047 26689780

[47] Williams HJ, Shepard ELC, Duriez O, Lambertucci SA. Can accelerometry be used to distinguish between flight types in soaring birds? Anim Biotelemetry 2015;3: 45 10.1186/s40317-015-0077-0

[48] Duriez O, Kato A, Tromp C, Dell’Omo G, Vyssotski AL, Sarrazin F, et al How cheap is soaring flight in raptors? A preliminary investigation in freely-flying vultures. PLoS One. 2014;9: e84887 10.1371/journal.pone.0084887 24454760 PMC3893159

[49] Shamoun-Baranes J, Bom R, Van Loon EE, Ens BJ, Oosterbeek K, Bouten W. From sensor data to animal behaviour: an oystercatcher example. PLoS One. 2012;7: e37997 10.1371/journal.pone.0037997 22693586 PMC3365100

[50] Larios DF, Rodríguez C, Barbancho J, Baena M, Leal MÁ, Marín J, et al An automatic weighting system for wild animals based in an artificial neural network: How to weigh wild animals without causing stress. Sensors. 2013;13: 2862–2883. 10.3390/s130302862 23449117 PMC3658719

[51] Rudolph SG. Foraging strategies of American kestrels during breeding. Ecology 1982;63: 1268–1276.

[52] Andersson M, Norberg RÅ. Evolution of reversed sexual size dimorphism and role partitioning among predatory birds, with a size scaling of flight performance. Biol J Linn Soc. 1981;15: 105–130.

[53] Ruppert D, Wand MP, Carroll RJ. Semiparametric regression. Cambridge, UK: Cambridge University Press; 2003.

[54] R Core Team. R: A language and environment for statistical computing. R Foundation for Statistical Computing, Vienna, Austria 2013.

[55] Wood SN. Fast stable restricted maximum likelihood and marginal likelihood estimation of semiparametric generalized linear models. J R Stat Soc. 2011;73: 3–36.

[56] Bates D, Maechler M, Bolker B, Walker S. lme4: Linear mixed-effects models using Eigen and S4. R package version 1.1–7. 2014.

[57] Pennycuick CJ. Modelling the flying bird. London: Academic Press; 2008.

[58] Sakamoto KQ, Sato K, Ishizuka M, Watanuki Y, Takahashi A, Daunt F, et al Can ethograms be automatically generated using body acceleration data from free-ranging birds? PLoS One. 2009;4: e5379 10.1371/journal.pone.0005379 19404389 PMC2671159

[59] Newton I. Population ecology of raptors. Black A, editor. London; 2010.

[60] Donázar JA, Negro JJ, Hiraldo F. Functional analysis of mate-feeding in the Lesser Kestrel *Falco naumanni*. Ornis Scand. 1992;23: 190–194.

[61] Cushman-Roisin B. Atmospheric Boundary Layer Environmetal Fluid Mechanics. New York: John Wiley & Sons; 2014.

[62] Vlachos C, Bakaloudis D, Chatzinikos E, Papadopoulos T, Tsalagas D. Aerial hunting behaviour of the lesser kestrel *Falco naumanni* during the breeding season in Thessaly (Greece). Acta Ornithol. 2003;38: 129–134.

[63] Withers PC. Aerodynamics and hydrodynamics of the “hovering” flight of Wilson’s storm petrel. J Exp Biol. 1979;80: 83–91.

[64] Rodríguez C, Tapia L, Kieny F, Bustamante J. Temporal changes in lesser kestrel (*Falco naumanni*) diet during the breeding season in southern Spain. J Raptor Res. 2010;44: 120–128.

[65] Berggren A. The effect of conspecifics on individual male movement in Roesel’s bush cricket, *Metrioptera roeseli*. Ecol Entomol. 2005;30: 480–483.

[66] Walker TJ. Effects of temperature, humidity, and age on stridulatory rates in *Atlanticus spp*. (Orthoptera: Tettigoniidae: Decticinae). Ann Entomol Soc Am. 1975;68: 607–611.

[67] Stiedl O, Bickmeyer U. Acoustic behaviour of *Ephippiger ephippiger* Fiebig (Orthoptera, Tettigoniidae) within a habitat of Southern France. Behav Processes. 1991;23: 125–135. 10.1016/0376-6357(91)90063-6 24897725

[68] Spiegel O, Getz WM, Nathan R. Factors influencing foraging search efficiency: Why do scarce lappet-faced vultures outperform ubiquitous white-backed vultures? Am Nat. 2013;181: 102–115. 10.1086/670009 23594555

[69] Wilson RP, Ropert-Coudert Y, Kato A. Rush and grab strategies in foraging marine endotherms: The case for haste in penguins. Anim Behav. 2002;63: 85–95.

[70] Wilson AM, Lowe JC, Roskilly K, Hudson PE, Golabek KA, McNutt JW. Locomotion dynamics of hunting in wild cheetahs. Nature 2013;498: 185–189. 10.1038/nature12295 23765495

[71] Penteriani V, Rutz C, Kenward R. Hunting behaviour and breeding performance of northern goshawks *Accipiter gentilis*, in relation to resource availability, sex, age and morphology. Naturwissenschaften. 2013;100: 935–42. 10.1007/s00114-013-1093-7 23995242

[72] Salamolard M, Weimerskirch H. Relationship between foraging effort and energy requirement throughout the breeding season in the wandering albatross. Funct Ecol. 1993;7: 643–652.

[73] Warrick DR, Tobalske BW, Powers DR. Aerodynamics of the hovering hummingbird. Nature 2005;435: 1094–1097. 10.1038/nature03647 15973407

[74] Norberg UML. Aerodynamics of hovering flight in the long-eared bat *Plecotus Auritus*. J Exp Biol. 1976;65: 459–470. 1003089 10.1242/jeb.65.2.459

[75] Ellington CP. The aerodynamics of hovering insect flight. I. The quasi-steady analysis. Philos Trans R Soc London Ser B. 1984;305: 1–15.

[76] Weimerskirch H, Le Corre M, Ropert-Coudert Y, Kato A, Marsac F. The three-dimensional flight of red-footed boobies: Adaptations to foraging in a tropical environment? Proc R Soc B. 2005;272: 53–61. 10.1098/rspb.2004.2918 15875570 PMC1634943

[77] Tella JL, Forero MG, Hiraldo F, Donázar JA. Conflicts between lesser kestrel conservation and European agricultural policies as identified by habitat use analyses. Conserv Biol. 1998;12: 593–604.

[78] Rodríguez C, Tapia L, Ribeiro E, Bustamante J. Crop vegetation structure is more important than crop type in determining where lesser kestrels forage. Bird Conserv Int. 2013;24: 438–452.

[79] Zank CM, Kemp AC. A comparison of hunting behaviour by lesser kestrels *Falco naumanni* and Eastern redfooted falcons *Falco amurensis* in their non-breeding south African range. Ostrich. 1996;67: 63–66.

[80] Mills GS. Foraging patterns of kestrels and shrikes and their relation to an optimal foraging model. PhD Thesis. University of Arizona, United States of America. 1979.

[81] Palatitz P, Solt S, Horváth É, Kotyman L. Hunting efficiency of red-footed falcons in different habitats. Ornis Hungarica. 2015;23: 32–47.

[82] Jin MS, Mullens T. A study of the relations between soil moisture, soil temperatures and surface temperatures using ARM observations and offline CLM4 simulations. 2014; 279–295.

