# Supplementary figures and images for: Combined use of tri-axial accelerometers and GPS reveals the flexible foraging strategy of a bird in relation to weather conditions
Source: PLoS One. 2017 Jun 7;12(6):e0177892. doi: 10.1371/journal.pone.0177892 (PMC5462363; doi:10.1371/journal.pone.0177892)

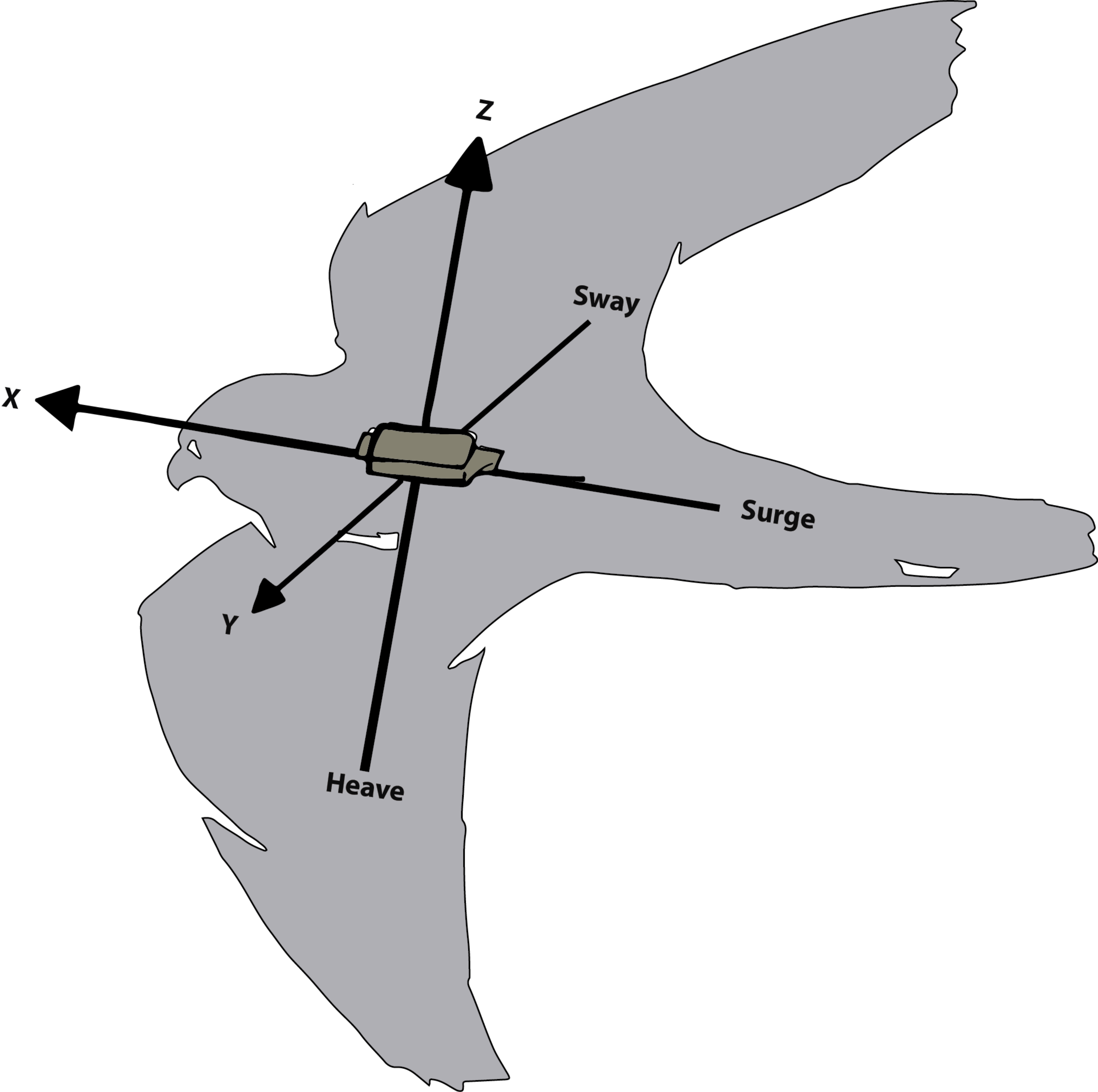

Supplement: S1 Fig — (TIF) [file pone.0177892.s001.tif]

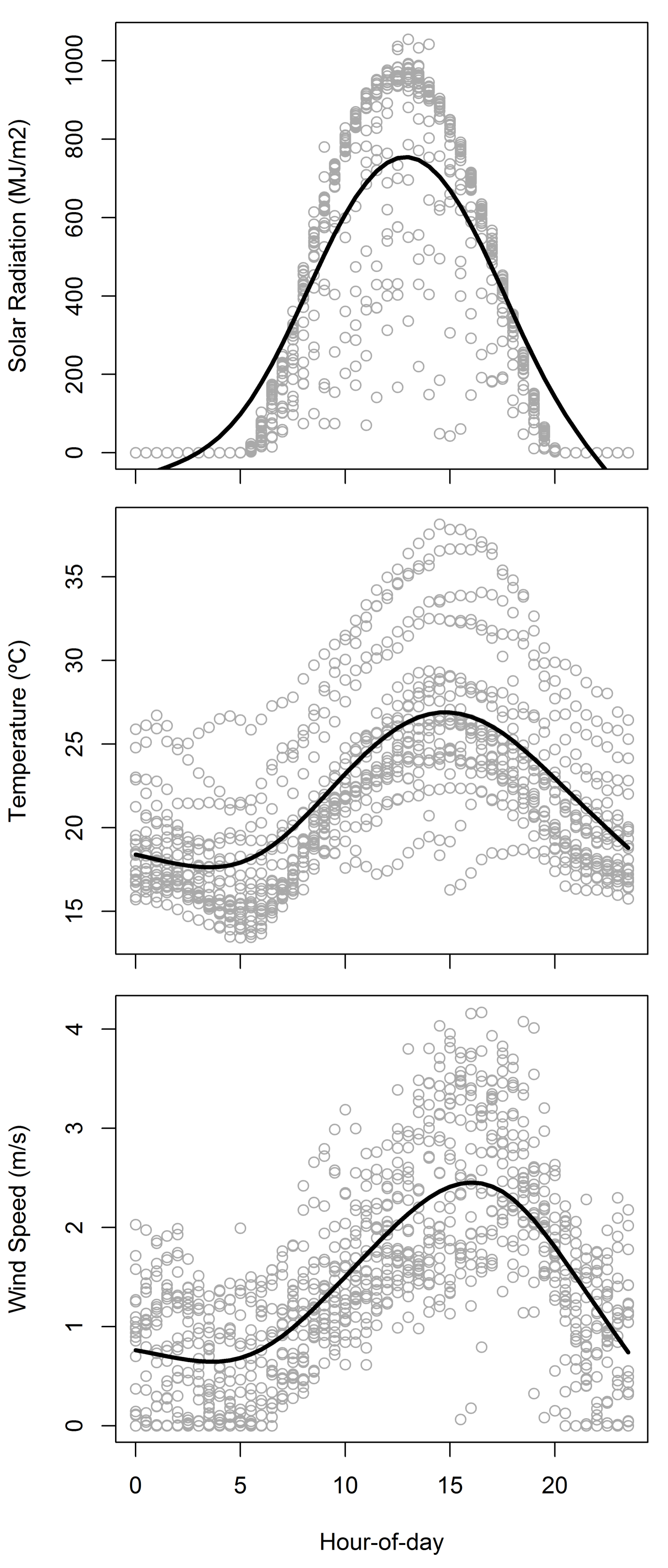

Supplement: S2 Fig — Sample size = 1,056 weather data samples from 22 days (3rd– 24th June). (TIF) [file pone.0177892.s002.tif]

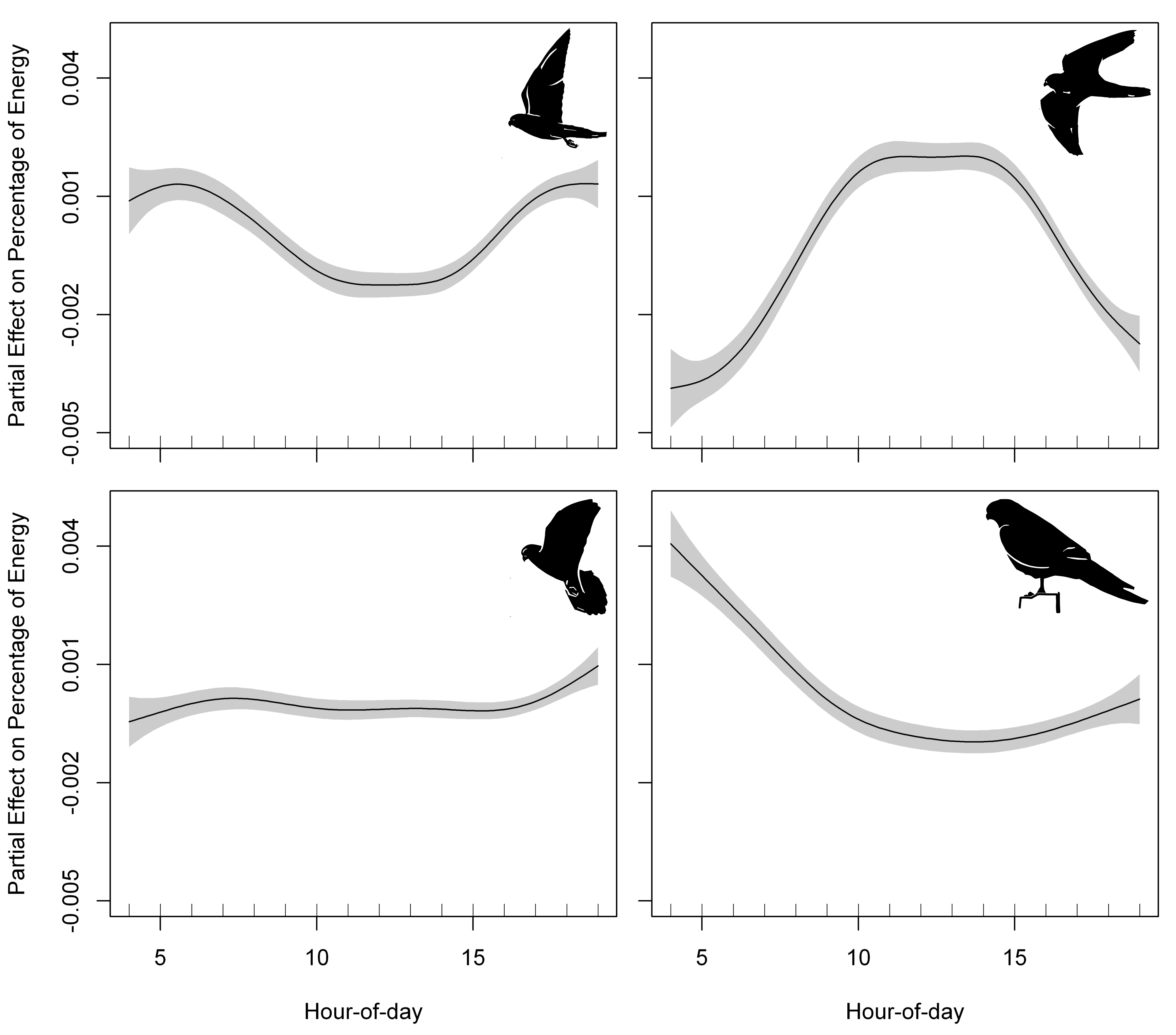

Supplement: S3 Fig — Flapping flight (upper left panel), soaring-gliding flight (upper right panel), hovering flight (bottom left panel) and perching (bottom right panel). Penalized smoothing splines of 6.29, 6.64, 4.67 and 4.20 degrees of freedom were adjusted to hour-of-day for flapping flight, soaring-gliding flight, hovering flight and perching, respectively. Grey shading represents the standard error of the mean effect. Sample size = 444 foraging trips. (TIF) [file pone.0177892.s003.tif]

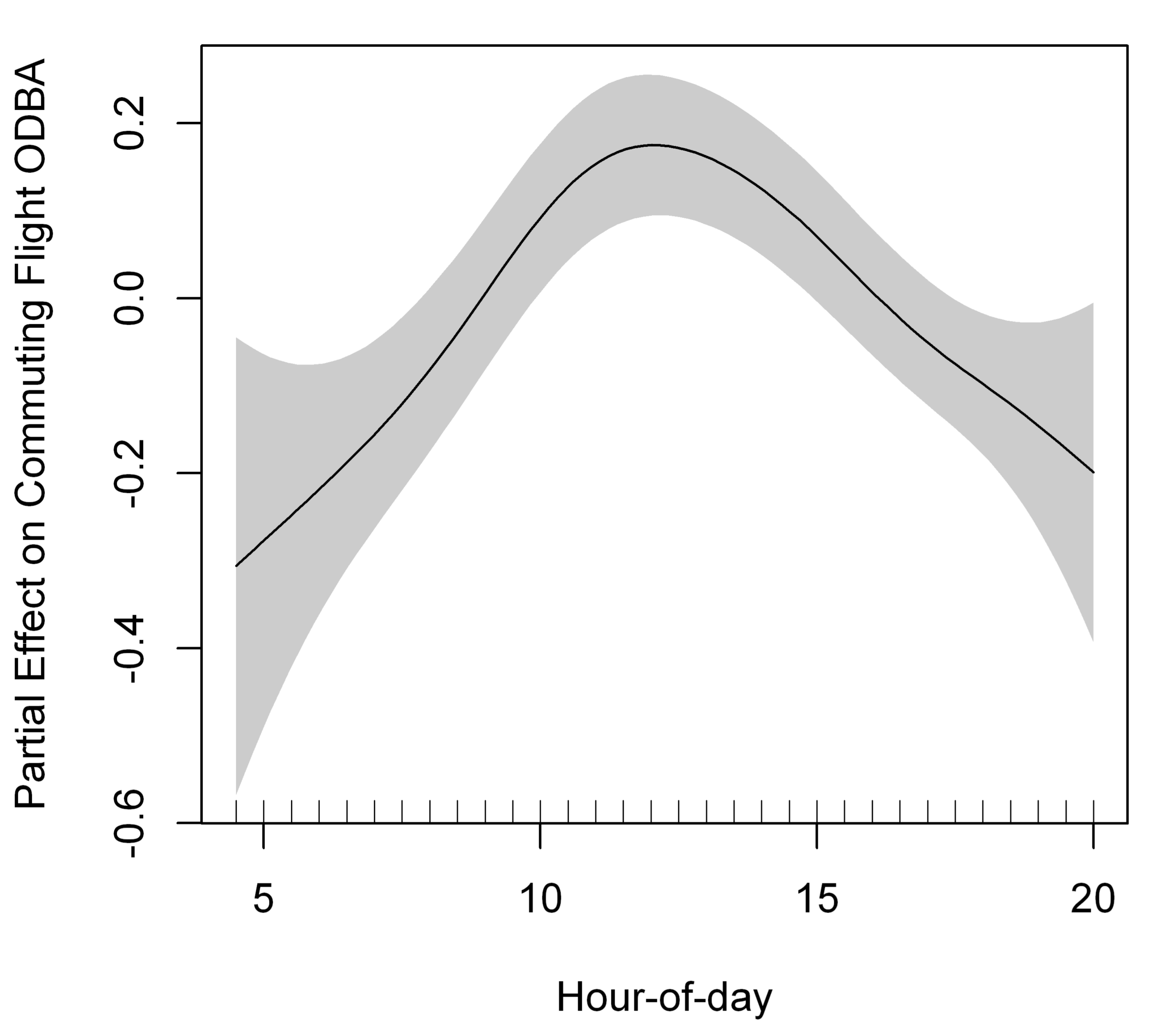

Supplement: S4 Fig — A penalized smoothing spline of 3.34 degrees of freedom was adjusted to hour-of-day. Grey shading represents the standard error of the mean effect. Sample size = 888 commuting flights. (TIF) [file pone.0177892.s004.tif]
