# Supplementary material for: Combined use of tri-axial accelerometers and GPS reveals the flexible foraging strategy of a bird in relation to weather conditions
Source: PLoS One. 2017 Jun 7;12(6):e0177892. doi: 10.1371/journal.pone.0177892 (PMC5462363; doi:10.1371/journal.pone.0177892)
Supplement: S1 Table — We built this matrix adding the results of the confusion matrix of each of the three models, which were built with the data of two of the three kestrels to classify behaviors of the third. Soaring-gliding and incubating/brooding are indicated as Gliding and Incubating, respectively. Observations correctly classified per behavior are shown in bold. (DOCX) [file pone.0177892.s005.docx]

|  |  | Predicted Behaviors | | | | | |
| --- | --- | --- | --- | --- | --- | --- | --- |
| Actual Behaviors |  | Flapping | Gliding / Incubating | Hovering | Perching | Total | **Recall** |
|  | Flapping | **3,622** | 48 | 163 | 18 | 3,851 | 94 % |
|  | Gliding / Incubating | 251 | **3,842** | 133 | 446 | 4,672 | 78 % |
|  | Hovering | 195 | 26 | **704** | 4 | 929 | 68 % |
|  | Perching | 12 | 152 | 10 | **2,474** | 2,648 | 93 % |
|  | Total | 4,080 | 4,068 | 1,010 | 2,942 | 12,100 | **Mean Recall** = 83% |
|  | **Precision** | 87 % | 94 % | 56 % | 81 % | **Mean Precision** = 80% | **Accuracy** = 88% **Kappa** = 83% |
