# Supplementary material for: Combined use of tri-axial accelerometers and GPS reveals the flexible foraging strategy of a bird in relation to weather conditions
Source: PLoS One. 2017 Jun 7;12(6):e0177892. doi: 10.1371/journal.pone.0177892 (PMC5462363; doi:10.1371/journal.pone.0177892)
Supplement: S2 Table — Statistically significant predictors are shown in bold: * p < 0.5, ** p < 0.01, *** p < 0.001. Sample size = 35 complete days of tracking. (DOCX) [file pone.0177892.s006.docx]

| **Predictors** | **Intercept** | **Sex**  (Female) | **Phenological Period**  (Incubation) |
| --- | --- | --- | --- |
| **Behaviors** | β ± S.E. (%) | β ± S.E. (%) | β ± S.E. (%) |
| Flapping | 53.33 ± 0.03 | **- 17.83 ± 0.07 **** | **- 16.19 ± 0.07** *** |
| Soaring-gliding | 28.79 ± 0.18 | **- 5.32 ± 0.06 *** | **- 7.96 ± 0.07** ** |
| Hovering | 12.52 ± 0.08 | - 0.86 ± 0.08 | 2.66 ± 0.04 |
| Perching | 5.02 ± 0.06 | **7.29 ± 0.16 *** | **8.51 ± 0.15** ** |
| Incubating/brooding | 0.99 ± 0.18 | **7.41 ± 0.48 *** | **11.40 ± 0.24** *** |
