# Supplementary material for: Combined use of tri-axial accelerometers and GPS reveals the flexible foraging strategy of a bird in relation to weather conditions
Source: PLoS One. 2017 Jun 7;12(6):e0177892. doi: 10.1371/journal.pone.0177892 (PMC5462363; doi:10.1371/journal.pone.0177892)
Supplement: S3 Table — Predictors included in the best model fitted to each variable as response are shown in bold.–indicates predictor not considered in the model. Sample Size = 444 foraging trips, 888 commuting flights, and 444 foraging events. (DOCX) [file pone.0177892.s007.docx]

| **Level of Analyses** | **Response Variable** | **Intercept** | **Sex**  (Female) | **Phenological Period** (Incubation) | **Commuting Flight** (Inwards) |
| --- | --- | --- | --- | --- | --- |
| Foraging Trip | Duration (min) | 36.28 ± 0.02 | 11.95 ± 0.02 | **45.35 ± 0.02** | - |
|  | Maximum Distance (km) | 2.98 ± 1.14 | **3.05 ± 1.26** | 0.99 ± 1.22 | - |
| Commuting Flights | Duration (min) | 6.60 ± 0.02 | 4.73 ± 0.02 | 0.58 ± 0.02 | **2.78 ± 0.02** |
|  | ODBA (g) | 152.22 ± 1.06 | **157.61 ± 1.11** | - 36.78 ± 1.14 | **94.16 ± 1.05** |
| Foraging Event | Duration (min) | 22.66 ± 0.02 | 5.10 ± 0.02 | **73.94 ± 0.02** | - |
|  | Hovering Ratio | 0.74 ± 0.65 | 0.001 ± 0.82 | **- 0.23 ± 0.71** | - |
|  | # Hovering Bouts | 8.81 ± 1.25 | 0.97 ± 1.62 | **7.86 ± 1.21** | - |
|  | # Perching Bouts | 3.87 ± 1.16 | 0.71 ± 1.35 | **11.84 ± 1.24** | - |
|  | # Hovering-Perching Bouts | 1.01 ± 1.20 | 0.50 ± 1.36 | **1.91 ± 1.27** | - |
