# Supplementary material for: Combined use of tri-axial accelerometers and GPS reveals the flexible foraging strategy of a bird in relation to weather conditions
Source: PLoS One. 2017 Jun 7;12(6):e0177892. doi: 10.1371/journal.pone.0177892 (PMC5462363; doi:10.1371/journal.pone.0177892)
Supplement: S4 Table — Statistically significant predictors are shown in bold. Sample size = 444 foraging trips. (DOCX) [file pone.0177892.s008.docx]

| **Predictors** | **β** | **S.E.** | **χ^2^** | **p-value** |
| --- | --- | --- | --- | --- |
| Intercept | 7,984.84 | 1.16 | - | - |
| Hour-of-day | - 7.02 | 1.01 | 0.01 | 0.91 |
| Sex (Female) | 4,741.13 | 1.29 | 2.54 | 0.28 |
| **Phenological Period** (Incubation) | 8,747.52 | 1.19 | 17.52 | < 0.001 |
