# Supplementary material for: Combined use of tri-axial accelerometers and GPS reveals the flexible foraging strategy of a bird in relation to weather conditions
Source: PLoS One. 2017 Jun 7;12(6):e0177892. doi: 10.1371/journal.pone.0177892 (PMC5462363; doi:10.1371/journal.pone.0177892)
Supplement: S5 Table — ΔAIC indicated the difference between the best model and the same model adding (negative values) or removing (positive values) the target predictor (depending on the predictors included in the best model). The higher the ΔAIC, the higher the importance of the predictor in the model fit. The predictors are classed as follows: Phenological Period as “PP”, individual sex as “S”, and hour-of-day as “H”. Hour-of-day was smoothed with a spline. Sample size = 444 foraging trips. (DOCX) [file pone.0177892.s009.docx]

| **Response Variables** | | **Best Model** ΔAIC = 0 | **PP** ΔAIC | **S** ΔAIC | **H** ΔAIC |
| --- | --- | --- | --- | --- | --- |
| Energy Expenditure | Flapping | H | - 7.95 | - 15.65 | 139.66 |
|  | Soaring-gliding | H | - 15.50 | - 14.30 | 339.14 |
|  | Hovering | H | - 11.80 | - 14.49 | 2.91 |
|  | Perching | H + PP | 4 | - 14.86 | 138.46 |
