# Supplementary material for: Combined use of tri-axial accelerometers and GPS reveals the flexible foraging strategy of a bird in relation to weather conditions
Source: PLoS One. 2017 Jun 7;12(6):e0177892. doi: 10.1371/journal.pone.0177892 (PMC5462363; doi:10.1371/journal.pone.0177892)
Supplement: S6 Table — Statistically significant predictors are shown in bold. Sample size = 444 foraging trips. (DOCX) [file pone.0177892.s010.docx]

| **Predictors** | **β** | **S.E.** | **χ^2^** | **p-value** |
| --- | --- | --- | --- | --- |
| Intercept | 660.11 | 1.19 | - | - |
| Hour-of-day | - 7.47 | 1.01 | 0.95 | 0.33 |
| Sex (Female) | 259.96 | 1.43 | 0.74 | 0.39 |
| **Phenological Period** (Incubation) | 1,629.88 | 1.36 | 19.24 | < 0.001 |
